# CuXBpy-MOF/TiO_2_ (X = I or Br) Composite Nanomaterials for Photocatalytic Hydrogen Generation

**DOI:** 10.3390/nano16191232

**Published:** 2026-09-30

**Authors:** Alisha Khan, Marie Le Pivert, Hiba EL Idrissi Bouyahyaoui, Md Faiz Alam, Diana Dragoe, Daniel Bahena Uribe, Alireza Ranjbari, Christophe Colbeau-Justin, Johnny Deschamps, Hynd Remita

**Affiliations:** 1 Institut de Chimie Physique (ICP), UMR 8000 CNRS, Université Paris-Saclay, 91405 Orsay, France; alisha.khan@universite-paris-saclay.fr (A.K.); marie.le_pivert@sorbonne-universite.fr (M.L.P.); hiba.el-idrissi@universite-paris-saclay.fr (H.E.I.B.); alireza.ranjbari@universite-paris-saclay.fr (A.R.); christophe.colbeau-justin@universite-paris-saclay.fr (C.C.-J.); 2 Laboratoire de Réactivité de Surface (LRS), UMR 7197 CNRS, Sorbonne Université, 75005 Paris, France; 3 Unité Chimie et Procédés (UCP), ENSTA Paris, Institut Polytechnique de Paris, 91120 Palaiseau, France; faiz.alam@ensta.fr; 4 Institut de Chimie Moléculaire et des Matériaux d’Orsay (ICMMO), UMR 8182 CNRS, Université Paris-Saclay, 91405 Orsay, France; diana.dragoe@universite-paris-saclay.fr; 5 Laboratorio Avanzado de Nanoscopía Electrónica (LANE), Centro de Investigación y de Estudios Avanzados del Instituto Politécnico Nacional (CINVESTAV), Mexico City 07360, Mexico; d.bahena.u@gmail.com

**Keywords:** hydrogen generation, photocatalysis, heterojunction, titanium dioxide (TiO_2_), metal-organic frameworks (MOFs), composite nanomaterials

## Abstract

Developing composites based on Metal-Organic Frameworks (MOFs) with enhanced photocatalytic properties for green hydrogen production offers a promising solution to energy and environmental challenges. This study focuses on the synthesis of copper-based MOFs (namely CuBrbpy and CuIbpy) combined with TiO_2_ to form heterojunction nanocomposites with improved photocatalytic activity for hydrogen generation. Triethylamine (TEA) was used as a hole scavenger and the synthesized materials were characterized by different techniques: ultraviolet-visible (UV-Vis) spectroscopy, Fourier-transform infrared (FTIR) spectroscopy, X-ray photoelectron spectroscopy (XPS), powder X-ray diffraction (XRD), transmission-electron microscopy (TEM), and photoelectrochemical techniques. Time-resolved microwave conductivity (TRMC) was used to investigate the charge-carrier dynamics. The photocatalytic hydrogen production rate of the nanocomposites was evaluated under UV-visible light irradiation and quantified using micro-gas chromatography (Micro-GC). The CuIbpy/TiO_2_ and CuBrbpy/TiO_2_ composites, with an optimized mass ratio of 1:20, exhibited the highest photocatalytic hydrogen evolution rates of 7.8 mmol g^−1^ h^−1^ and 7.0 mmol g^−1^ h^−1^, respectively. The enhanced photocatalytic activity is mainly associated with improved interfacial charge transfer and charge-carrier separation due to the MOF/TiO_2_ heterojunction. Cycling experiments showed a slight decrease of activity probably due to copper and nitrogen leaching, as confirmed by post XPS analysis. These findings also demonstrate that the ligand environment significantly governs the electronic interactions and photocatalytic behaviour of Cu-MOF/TiO_2_ composite systems.

## 1. Introduction

The continuous increase in global energy consumption and its associated environmental consequences have intensified the search for sustainable energy alternatives. Fossil fuels account for more than 85% of the global energy consumption, and their combustion leads to large quantities of carbon dioxide (CO_2_) emissions [1], which have important consequences for climate change. Therefore, renewable energies should be a viable course of action in the near future. However, even in the current state of knowledge, the various resources (solar, wind, hydropower, geothermal, and biomass) remain subject to technological constraints, such as intermittency. To circumvent this problem, energy vectors seem to be an appropriate solution, and hydrogen (H_2_) emerges as a promising candidate among known energy carriers.

In contrast to carbon-based fuels, hydrogen can be considered as an environmentally friendly energy source when it is produced using raw materials for green energy. It possesses a high lower heating value (LHV) of 120 MJ kg^−1^, which is significantly greater than gasoline’s 43 MJ kg^−1^, and has multiple advantages as a chemical fuel [2]. It is a clean energy vector and, except for small amounts of nitrous oxides, does not produce pollutants such as carbon dioxide (CO_2_), carbon monoxide (CO), or hydrocarbons during combustion in air. However, the most important part of hydrogen is currently mainly produced from fossil fuels through steam reforming of fossil energies, a process related to significant CO_2_ emissions. It is then necessary to produce green hydrogen with renewable energy sources, which account currently for only ~1% of global H_2_ production [3]. Solar energy, often regarded as ethical and sustainable, could be a viable option due to its inexhaustibility and widespread availability. Moreover, solar-driven water splitting is one of the most promising ways to produce green fuel by converting solar energy into hydrogen (STH), and, among the various solar energy conversion techniques, photocatalytic water splitting is one of the simplest ways, which can be viewed as a biomimetic approach to an artificial photosynthesis system, to produce green H_2_ with carbon-free emissions [4].

In a photocatalysis process, when the energy of the photons is equal to or higher than the band gap, the semiconductor harvests incident light to produce electrons and holes. These charge carriers (electrons and holes), when they escape recombination, can migrate to the surface of the photocatalyst and participate in redox reactions such as the reduction of H^+^ to produce H_2_ or oxidation of electron donor species, also called hole scavengers. However, a major fraction of the charge carriers (electrons and holes) recombines in the bulk of the photocatalyst or undergoes surface recombination before inducing redox reactions, thereby reducing the quantum yield and, consequently, the photocatalytic activity.

Therefore, the separation of these charge carriers represents the first major challenge in photocatalysis. Most inorganic semiconductors used as photocatalysts (such as TiO_2_ or ZnO) have a large band gap and are therefore active only under UV light, which accounts for approximately 4–5% of the solar spectrum. The second challenge in photocatalysis is therefore the development of photocatalysts that harvest solar light more efficiently and are active under visible light. Visible-light-responsive organic semiconductors, such as conjugated polymer nanostructures, have been explored [5,6] and more recently, metal-organic frameworks (MOFs) materials which are crystalline porous materials consisting of metal ions or metal clusters linked by organic ligands, have become a promising type of semi-conductor, garnering significant attention in photocatalysis for solar fuels generation [7,8,9,10,11]. More precisely, Cu-based MOFs materials emerge as very promising for H_2_ generation [7,12,13]. In our previous work, we developed very active composite photocatalysts HKUST-1/TiO_2_ for hydrogen generation [7,12,13], and the best photocatalytic activity (5.1 mmol g^−1^ h^−1^) was obtained with HKUST-1/TiO_2_ (1:20 mass ratio). Furthermore, this photocatalyst exhibited improved activity with cycling, and after six photocatalytic cycles, the hydrogen generation rate increased to 13.2 mmol g^−1^ h^−1^, surpassing 3 wt% CuO (TiO_2_) (4.1 mmol g^−1^ h^−1^) and the benchmark 1wt % Pt (TiO_2_) (7.9 mmol g^−1^ h^−1^) materials (1 nm-Pt nanoparticles induced by radiolysis on titania) [13], tested in an identical reactor and under the same xenon lamp irradiation conditions. It was then demonstrated that the HKUST-1/TiO_2_ (1:20 mass ratio) composite remains highly photoactive for hydrogen generation through charge carrier delocalization leading to different copper oxidation states stabilized by the ligand [13]. More generally, the coupling of two or more components within a single material has become a common route to enhance catalytic performance, since the synergy between the coupled components modulates the electronic structure of the active sites and promotes charge transfer [14,15,16]. Based on these results, we were motivated to explore composite materials based on TiO_2_ coupled with other copper-based MOFs like CuXbpy (X = I or Br) MOFs known for their stability, low-cost materials composition, and ease of synthesis. The MOF-TiO_2_ composite materials are usually centered on porous MOF materials which require solvo-thermal synthesis in autoclave and activation (heating under vacuum or supercritical drying) techniques. These technologies rely on specific and expensive equipment, and also require energy and time. The CuXbpy/TiO_2_ (X = I or Br) MOFs, linked with a 4,4′ bipyridine (bpy) ligand, are noble metal free-materials synthesized in a beaker and washed with water, at ambient temperature and atmospheric pressure, in less than one hour. Moreover, the materials also possess a network of microporous channels which avoid the intrusion of guest molecules and then the activation phase after synthesis [17]. It therefore offers a novelty and a distinctiveness in terms of energy savings, as well as ease and time of synthesis. In addition, Pristine CuIbpy MOF has already been studied in the literature as photocatalyst and proved to be promising for hydrogen production [18]. Related studies based on this CuXbpy (X = I or Br) MOF coupled with coordination polymers (CPs) [19], cerium oxide (CeO_2_) [20], or using anthracene-based bipyridine as ligand [21] have also been successfully conducted in the context of photocatalytic hydrogen production. In another recent study, the photocatalytic activity of Cu_2_O@CuX-bpy (X = Cl, Br, I) was explored, and it was found that this composite results in high photocatalytic performance in reducing CO_2_ to formate (HCOOH) [22]. It appears then clearly that the association of CuXbpy (X = I or Br) MOF frameworks with semiconductors can significantly improve charge separation and photocatalytic activity. A coupling with titanium dioxide (TiO_2_) to form CuIbpy/TiO_2_ and CuBrbpy/TiO_2_ heterojunctions could be then very promising and has not been investigated yet. Therefore, the present work focuses on the synthesis, characterization, and photocatalytic study of CuIbpy/TiO_2_ and CuBrbpy/TiO_2_ composites, with particular emphasis on understanding the influence of the halide ligand and the effect of CuXbpy (X = I or Br)/TiO_2_ heterojunction formation on charge-carrier dynamics and hydrogen generation evolution.

The structure of CuBrbpy MOF has been previously reported [17]. In this framework, Cu_2_(µ_2_-Br)_2_ clusters are linked by 4,4′-bipyridine bridges and each Cu ion has a tetrahedral geometry, coordinating to two µ_2_-Br ions and two bridging bpy ligands. As a result, each CuBr dimer is coordinated to four bpy bridges. It is to be noted that the structures of CuIbpy and CuBrbpy MOFs are isomorphous [17].

## 2. Materials and Experimental Procedures

### 2.1. Materials

Commercial P25 titanium (IV) dioxide, with a specific surface area of 50 m^2^ g^−1^, composed of a mixture of crystalline phases (anatase 73–85%, rutile 14–17%) and amorphous Titania (0–13%), [23] was purchased from EVONIK (Essen, Germany). Triethylamine (TEA, N(CH_2_CH_3_)_3_), 4,4′-Bipyridine (bpy, 4-(C_5_H_4_N)-4-(C_5_H_4_N)), Copper (I) iodide (CuI), Copper (I) bromide (CuBr), sodium sulfate (Na_2_SO_4_, ≥99.0%), 2-Propanol ((CH_3_)_2_CHOH, 99.5%), Fluorine Doped Tin Oxide (FTO) and Nafion (~5% in a mixture of lower aliphatic alcohols and water) were purchased from Sigma Aldrich (Saint-Quentin-Fallavier, France). Gold lacey carbon grids (200 mesh) were purchased from Agar Scientific (Rotherham, UK). Ultrapure water (Millipore system, 18.6 MΩ cm) obtained from PURELAB Quest (ELGA LabWater, High Wycombe, UK) was used throughout the experiments. Nitrogen gas (N_2_, ALPHAGAZ^TM^ 2, ≥99.9999%) was supplied by Air Liquide (Paris, France). All the reagents were analytically pure and used without further purification.

### 2.2. Synthesis of CuBrbpy and CuIbpy Metal-Organic Framework Materials (MOFs)

CuBrbpy and CuIbpy MOF materials were synthesized using a previously reported simple method [17]. The synthesis process is schematically represented in Figure S1, Supporting Information.

In a typical experiment of CuBrbpy MOF synthesis, 190 mg of copper bromide (CuBr) were dissolved in 100 mL of acetonitrile (Soln. A). Then, 210 mg 4,4′-bipyridine were dissolved in 100 mL of acetonitrile (soln. B). Soln. A and soln. B were then mixed in a beaker, and the mixed solution was stirred for 50 min at 300 rpm. A change in color was observed from yellow to thick red plates. Once the stirring was complete, the mixture underwent a filtration process with a glass frit of porosity 5. The product was then filtered and washed three times with 10 mL of acetonitrile and three times with 10 mL of water. After filtration, the compound was dried overnight in an oven set to a temperature of 30 °C. The obtained yield was 80%.

The synthesis procedure of CuIbpy MOF was similar to that of CuBrbpy MOF (Figure S1, Supporting Information). For this material, 400 mg of copper iodide (CuI) were dissolved in 100 mL of acetonitrile (soln. A), and 660 mg of 4,4′-bipyridine were dissolved in 100 mL of acetonitrile (soln. B). Then, soln. A and soln. B were mixed together. Finally, thick red plates were obtained with a yield of 55%.

In the initial procedure of synthesis used, as reported in the literature [17], the two MOFs materials were washed with diethyl ether. We then decided to replace this organic solvent with water to be more environmentally friendly. Some tests of washing with diethyl ether were performed, and no significant differences in terms of photocatalytic activity or hydrogen generation were observed.

### 2.3. Synthesis of Composites of CuXbpy (X = I, Br) MOFs Materials with TiO_2_

**Figure 1:**
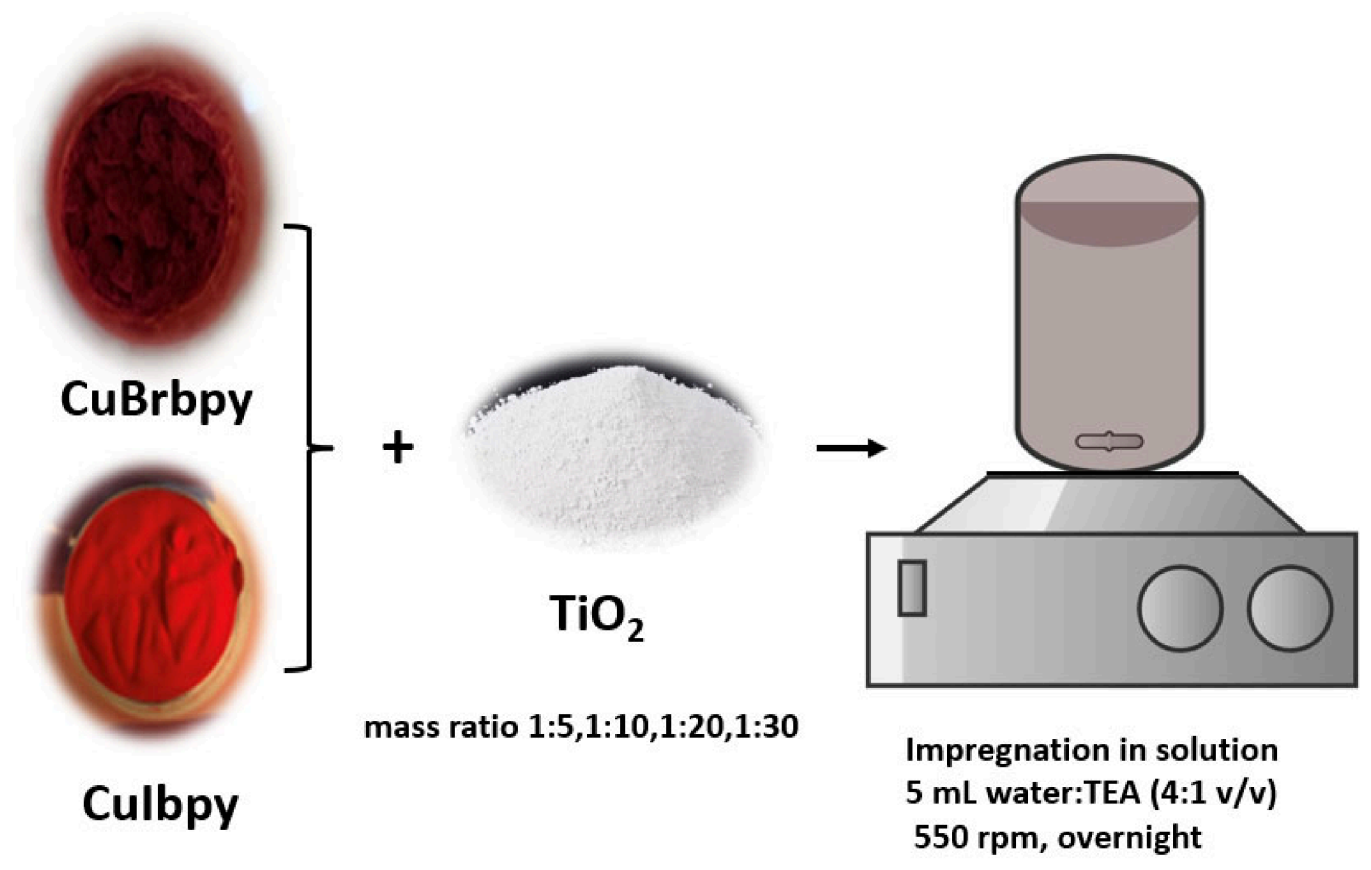
Schematic representation of the preparation of the CuXbpy/TiO_2_ (X = I or Br) composites by impregnation.

In one of our previous studies on CuMOF/TiO_2_ composites, we compared an in situ solvo-thermal synthesis approach in which HKUST-1 was grown in the presence of TiO_2_ with the composite prepared by impregnation in a solution of commercial HKUST-1 and TiO_2_ in methanol. It was expected that in situ HKUST-1/TiO_2_ would form a junction, which can lead to better charge carrier separation. However, under similar photocatalysis conditions, the composite prepared by wet impregnation showed better results than the in situ approach. This reduced activity of the in situ HKUST-1/TiO_2_ (1:20) composite could be due to less copper nanocluster formation, as observed in TEM, TiO_2_ aggregation detected by SEM, or due to a lower amount of copper content in the final in situ composite [13]. Based on these findings, we selected the impregnation in solution technique for the preparation of the CuXbpy/TiO_2_ composite presented in this manuscript. The composites of CuXbpy (X = I or Br) MOFs with TiO_2_ (with different mass ratio 1:5, 1:10, 1:20, 1:30) were therefore prepared by impregnation in solution by simple overnight mixing at 550 rpm in a 5 mL solvent mixture of water:TEA (4:1 volume ratio) (Figure 1). This overnight mixing is necessary to ensure effective bonding between CuXbpy (X = I or Br) MOFs and TiO_2_.

### 2.4. Characterization of Photocatalysts

To correlate structure, properties, and photocatalytic activity, composites were characterized by different methods involving the investigation of their optical properties, morphology, crystallographic structure, intermolecular interactions, oxidation states, and mechanistic study. The optical properties of the sample were investigated using an Agilent technologies Cary 5000 UV-Vis-NIR spectrophotometer (Santa Clara, CA, USA), equipped with a Praying Mantis diffuse reflectance accessory (Harrick Scientific Products, Pleasantville, NY, USA) over the wavelength range of 200–800 nm. Barium sulfate (BaSO_4_) was used as the reference for setting the maximum reflectance standard, and calibrated to 100% reflectance. The optical band gap energies were calculated from diffuse reflectance spectra using the Kubelka–Munk function F(R∞) (Equation (1)), which converts measured reflectance spectra to corresponding absorption spectra.

F(R_∞_) = K/S = (1 − R_∞_)^2^/2R_∞_ (1)

where, R_∞_ = (R_sample_/R_standard_) is the reflectance of an infinitely thick specimen, and K and S are the absorption and scattering coefficients, respectively [24].

The band gaps of the samples can then be determined by using Equation (2).

(F(R_∞_).hv)^1/γ^ = B(h𝛎 − E_g_) (2)

where h is the Planck constant, *ν* is the photon’s frequency, B is a energy independent constant, and E_g_ is the band gap energy [25]. The factor γ depends on the nature of the electron transition and is equal to 1/2 or 2 for the direct and the indirect transition bandgaps, respectively [26]. The bandgap energies of the samples were calculated by considering the indirect transition of the anatase phase of TiO_2_ [26,27,28] for the pristine samples of TiO_2_ and their composites with CuBrbpy and CuIbpy.

Thermogravimetric analyses were investigated using a Setaram LabSys evo TGA-DTA system starting from 20 to 850 °C at a heating rate of 5 °C min^−1^ under argon flow of 30 mL min^−1^. The sample was subsequently cooled to room temperature under the same gas flow, and the cooling rate was 20 °C min^−1^.

TEM samples were prepared by dispersing a few milligrams of the sample in ethanol and ultrasonically treating it for a few tens of seconds. The suspension was dropped onto 200-mesh gold-lacy carbon grids. The high-angle annular dark-field scanning transmission electron microscopy (HAADF-STEM) images of the samples were acquired using a C_s_-corrected JEOL-ARM6200F electron transmission microscope (JEOL ltd, Tokyo, Japan). To study the elemental composition of the samples, EDS mapping was performed using an Oxford instrument (Oxford Instrument, High Wycombe, UK) with a solid-state detector and an 80 mm^2^ surface window.

Powder XRD was recorded with an Panalytical (Malvern Panalytical, Almelo, The Netherlands) X’Pert diffractometer and a Cu Kα radiation source with wavelength λ = 1.5406 A°.

FTIR spectra of the samples were obtained between 600–4000 cm^−1^ with a Bruker Vertex 70 FTIR spectrometer (Bruker Optics, Ettlingen, Germany) equipped with a diamond ATR apparatus (PIKEMIRACLE crystal plate, diamond/ZnSe; PIKE Technologies, Madison, WI, USA) and a MCT detector. For each measurement, the samples were placed on a clean ZnSe diamond crystal, and a minimum of 36 scans were performed at a resolution of 2.0 cm^−1^.

XPS measurements were conducted using a K Alpha spectrometer (Thermo Fischer Scientific, Hillsboro, OR, USA), equipped with a hemispherical electron energy analyzer and a monochromatic X-ray source Al K_α_ (1486.6 eV). Spectra were collected from an irradiated area of approximately 1 mm^2^, obtained using a 400 µm X-ray spot size. All spectra were calibrated against the Ti2p_3/2_ peak set at 458.6 eV as a standard reference, and the analyzer precision was ±0.2 eV. The peak-fitting routine included a Shirley-type background and Gauss–Lorentz line shapes with 30% of Lorentzian character. Survey spectra were measured with a pass energy of 100 eV, an energy step of 1 eV, and a 10 ms dwell time. The narrow scans were measured with a pass energy of 50 eV, an energy step of 0.1 eV, and a dwell time of 50 ms, covering the binding energy range from 0 to 1350 eV. Elemental quantification was based on peak area integration after subtracting the background, and peak fitting was performed using Thermo Avantage software (6.7.0). For determining elemental composition, relative sensitivity factors (7.91, 2.93, 1.00, 2.729, and 16.73 for Ti 2p, O 1s, C 1s, Br 3d, and Cu 2p, respectively), obtained from instrument-modified Schofield cross-sections, were applied.

It is important to note that the XPS analysis was not conducted on pristine CuIbpy MOF because iodine is a well-known contaminant in vacuum chambers.

### 2.5. Photoelectrochemical Measurements and TRMC Studies

Photoelectrochemical measurements were measured using a Metrohm Autolab PGSTAT204 electrochemical station (Metrohm Autolab B.V., Utrecht, The Netherlands) operated with Nova 2.1 software with a three-electrodes configuration. A photocatalyst-coated FTO (Fluorine Doped Tin Oxide), a Pt foil, and Ag/AgCl (saturated KCl) were employed as working, counter, and reference electrodes, respectively. The working electrode was prepared by dispersing 2 mg of the sample in 1 mL isopropanol and 40 µL Nafion. Subsequently, 100 µL of the resulting suspension was drop-casted onto an FTO substrate (1.5 × 2.5 cm), followed by overnight drying at room temperature before use. For transient photocurrents and electrochemical impedance spectroscopy (EIS), 0.5 M sodium sulfate (Na_2_SO_4_) aqueous solution was used as an electrolyte in a quartz electrochemical cell. A solar simulator (LCS-100, Newport Corporation/Oriel instruments Irvine, CA, USA) was used as the irradiation source. The photocurrent density-time curves were collected at a constant potential of 0 V under intermittent illumination with light switched on/off every 30 s intervals.

The charge carrier dynamics, a crucial factor influencing the photocatalytic activity, was studied by the time-resolved microwave conductivity (TRMC) technique for all the prepared samples. This technique measures the relative change (∆P(t)) in the microwave power upon laser excitation of the sample [29,30,31,32]. A pulsed and tunable Nd:YAG laser source (200–2000 nm) coupled with an optical OPO:EKSPLANT 342B oscillator was used to irradiate the sample, with excitation wavelengths of 380 nm (UV) and 450 nm (visible). Microwaves were generated using a Gunn diode at a frequency of 29–31 GHz.

According to Equation (3), the relative change in the microwave power reflected by the sample is associated with small variations of the sample conductivity:

ΔP(t)/P = AΔσ(t) = Ae ∑_i_ Δn_i_(t)μ_i_ (3)

where Δn_i_(t) corresponds to the density of free electrons at time t, µ_e_ is their respective mobility, A is a time-independent sensitivity factor which relies on microwave conductivity and the semiconductor conductivities [33,34].

In the case of TiO_2_, the TRMC signal is mainly attributed to the number of free electrons as the mobility of electrons is much higher than that of the holes [35]. In a TRMC signal for TiO_2_, a high I_max_ typically reflects efficient charge carriers’ generation and a high number of electrons in the CB of TiO_2_, as electrons are more mobile than holes in titania. The subsequent decay in the signal is due to the recombination or trapping.

### 2.6. Photocatalytic Tests for Hydrogen Generation

The photocatalytic activity of the samples for hydrogen generation from a triethylamine-water mixture, using TEA as a hole scavenger, was studied in a closed quartz reactor manufactured by Peschl Ultraviolet GmbH (Mainz, Germany) (headspace 17 mL, dimensions illustrated in Figure S2, Supporting Information) with a 500 W Hg lamp (Supplied by Helios quartz, Milano, Italy). The radiation spectrum of the mercury lamp is shown in the Figure S3 (Supporting Information). This mercury lamp emits across the entire UV spectrum, including UV A, UV B, and UV C, with an intense emission at 366 nm in the UV A range. We dispersed 6.66 mg of the photocatalyst in 20 mL of degassed water-TEA mixture solution, with a V_TEA_/V_water_ ratio of 1/19. The concentration, the 500 W Hg lamp irradiation, and TEA amount (5 vol%) were selected to match the experimental conditions of the studies reported in the literature for pristine CuXbpy (X = I or Br) MOFs. Applying the same parameters allows a meaningful comparison of the photocatalytic cycles. The reactor was sealed with a septum, and the oxygen was purged from the reactor by bubbling the suspension with nitrogen for 25 min. Finally, the photocatalytic activity of the sample was measured under Hg lamp (500 W) for four hours under stirring at 300 rpm. Hydrogen production was quantified using INFICON Micro GC Fusion (INFICON, East Syracuse, NY, USA). Gas samples were collected from the reactor headspace every hour using a gas syringe and injected into the micro-GC to measure and quantify the amount of H_2_ produced.

Photocatalytic activity tests with cycling were performed using 6.66 mg of the fresh photocatalyst (CuXbpy/TiO_2_ mass ratio 1:20; X = Br, I) dispersed in 20 mL of TEA/water mixture (V_TEA_/V_water_ = 1/19). The reactor was sealed and purged with nitrogen for 25 min, followed by irradiation for 4 h in a Helios quartz photoreactor. After each photocatalytic run, the photocatalyst was then recovered by centrifugation and dried overnight at 80 °C. The next day, the recovered photocatalyst was weighed and redispersed in a fresh 5 vol% TEA aqueous solution, maintaining a concentration of 0.33 mg mL^−1^. The same procedure was repeated for three consecutive photocatalytic cycles.

## 3. Results and Discussions

### 3.1. Characterization of CuIbpy and CuBrbpy MOF

#### 3.1.1. Thermogravimetric Analyses (TGA)

TGA analysis for both pristine CuIbpy and CuBrbpy MOF materials, before and after activation, is shown in Figure S4, Supporting Information. There was almost no difference in the mass loss before and after activation, indicating that these MOFs do not require any activation. A simple drying process was enough to remove the solvent from the external surface of the MOF, as the small aperture of the MOF pore channels prevents organic solvent penetration into the pores [17]. In the case of CuIbpy (as shown in Figure S4a, Supporting Information), an initial mass loss of 24% is observed between 215 °C and 280 °C, while for Cu-Br-bpy (Figure S4b, Supporting Information), a loss of 27% is observed between 220 °C and 300 °C. At this temperature, CuIbpy and CuBrbpy likely decompose into [(CuI)_2_(bpy)] and [(CuBr)_2_(bpy)], respectively.

A second loss of 23% (Figure S4a, Supporting Information) occurs between 330 °C and 400 °C for CuIbpy, while a loss of 28% (Figure S4b, Supporting Information), between 355 °C and 490 °C, occurs for CuBrbpy. At these temperatures, [(CuI)_2_(bpy)] and [(CuBr)_2_(bpy)] are further decomposing into [(CuI)_2_(bpy)_1/2_] and [(CuBr)_2_(bpy)_1/2_], and subsequently into CuI and CuBr. The third mass loss reached 52% for CuBrbpy between 490 °C and 815 °C (Figure S4a, Supporting Information), and 43% for CuIbpy between 490 °C and 795 °C (Figure S4b, Supporting Information). At such high temperatures, the remaining structure is likely to be decomposed, probably leading to the formation of copper oxides or copper metal [36,37].

#### 3.1.2. FTIR, UV-Vis DRS, and XRD Analysis of CuIbpy and CuBrbpy

**Figure 2:**
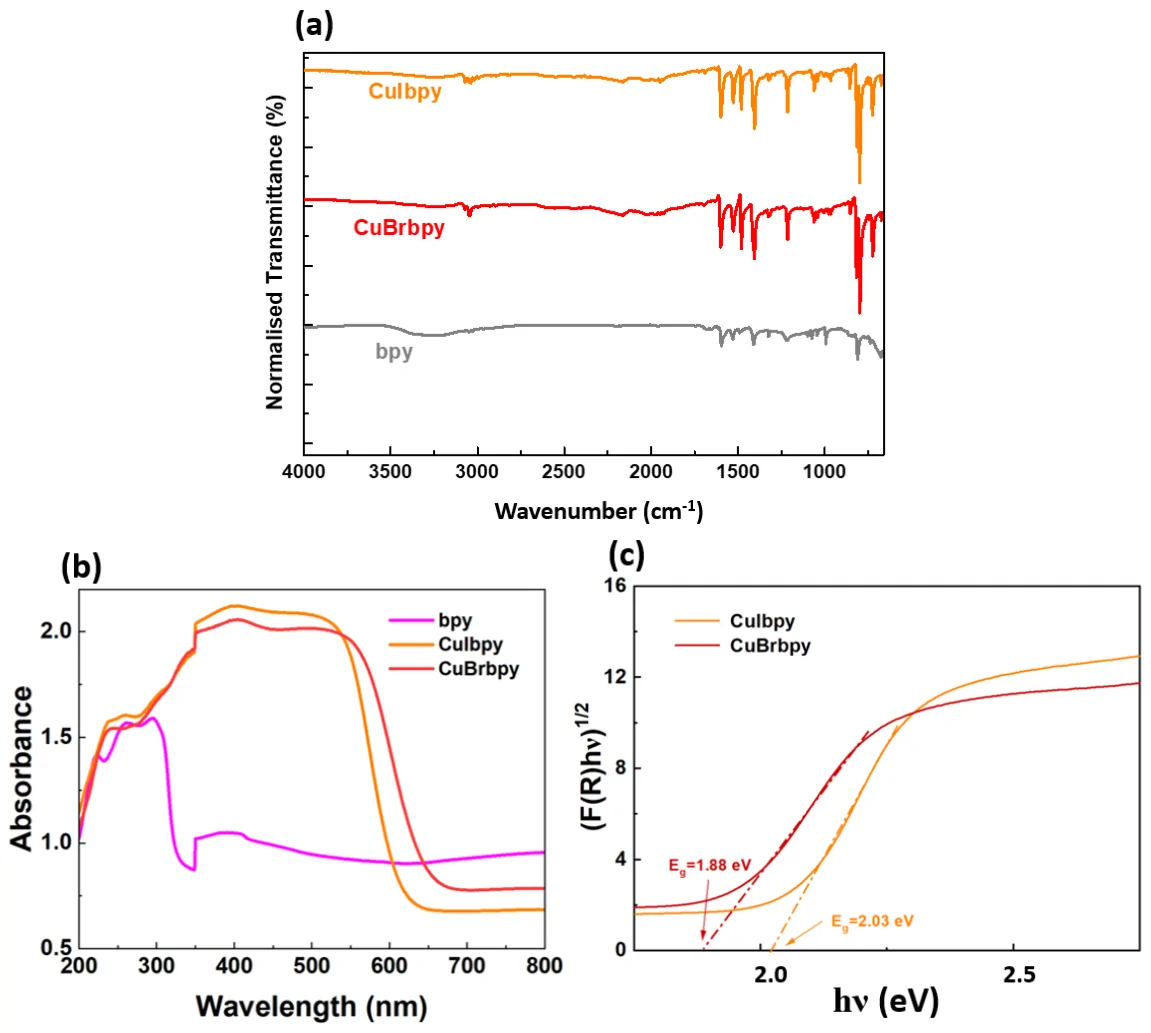
FTIR spectra of pristine CuIbpy and CuBrbpy MOFs compared to the bpy ligand (**a**), Comparison of UV-visible DRS of bpy with pristine CuIbpy and CuBrbpy MOFs (**b**) Kubelka-Munk absorption spectra indicating band gaps of pristine CuIbpy and CuBrbpy MOFs (**c**).

In Figure 2a, the FTIR spectrum of bpy is compared with the ones of CuIbpy and CuBrbpy. The bipyridine ligand displays multiple distinct characteristic IR bands, primarily due to the vibrations of the aromatic C-H, C=C, and C=N bonds. The bipyridine bands include aromatic C-H (stretching) at 3000–3100 cm^−1^ [38], and aromatic ring stretching vibrations of coupled C=C and C=N character at approximately at 1400–1615 cm^−1^, which arise as ring modes of the delocalized π-system rather than as separate bond stretches [39,40]. Out-of-plane and in-plane C-H bending is frequently seen in the 1300–600 cm^−1^ range. The characteristic bipyridine bands are retained in both CuIbpy and CuBrbpy, confirming that the ligand remains intact upon complex formation. Small but systematic shifts in these band positions are observed relative to free bpy [41,42]. The band at 1595 cm^−1^ shifts slightly upwards, to 1598 and 1600 cm^−1^ in CuIbpy and CuBrbpy, respectively; comparable upward shifts of ligand ring modes have been reported for M(4,4′-bipy)Cl_2_ complexes and attributed to mechanical coupling with the low-frequency metal–nitrogen stretching vibration [43]. The remaining bands shift to lower wavenumbers, most notably those at 1224, 809, and 737 cm^−1^, which move by 9–16 cm^−1^ [44]. These shifts substantially exceed the spectral resolution of 2.0 cm^−1^ and are reproduced consistently in both complexes, confirming coordination of the pyridyl nitrogen atoms to the Cu(I) center.

Coordination of the ligand to the metal can induce shifts in the positions of the C=N stretching modes due to the interactions between Cu(I) and nitrogen atoms of bpy ligands towards lower wavenumbers.

UV-Vis DRS spectra of pristine CuIbpy and CuBrbpy MOFs, compared to the bpy ligand, are shown in Figure 2b. It is clearly observed that the absorption bands of CuIbpy and CuBrbpy exhibit a significant red-shift compared to bpy ligand. These shifts are related to the influence of coordinated Cu^+^ ions on the excited state of the ligand, d-d transitions of Cu^+^ ions, and π-π * stacking of bpy ligands [45]. The band gap for the CuIbpy and CuBrbpy MOFs was also calculated from Kubelka–Munk spectra (Figure 2c) and found to be 2.03 eV and 1.88 eV, respectively, which is consistent with the literature values, 2.00 eV for CuIbpy and 1.90 eV for CuBrbpy [18].

The simulated powder X-ray diffraction (PXRD) [17] patterns of CuIbpy and CuBrbpy MOFs align with the experimental ones (Figure S5, Supporting Information).

Finally, all these characterization results prove a successful synthesis of the CuIbpy and CuBrbpy MOFs.

### 3.2. Characterization of the TiO_2_-MOF Composites

#### 3.2.1. UV-Vis DRS of the TiO_2_-MOF Composites

**Figure 3:**
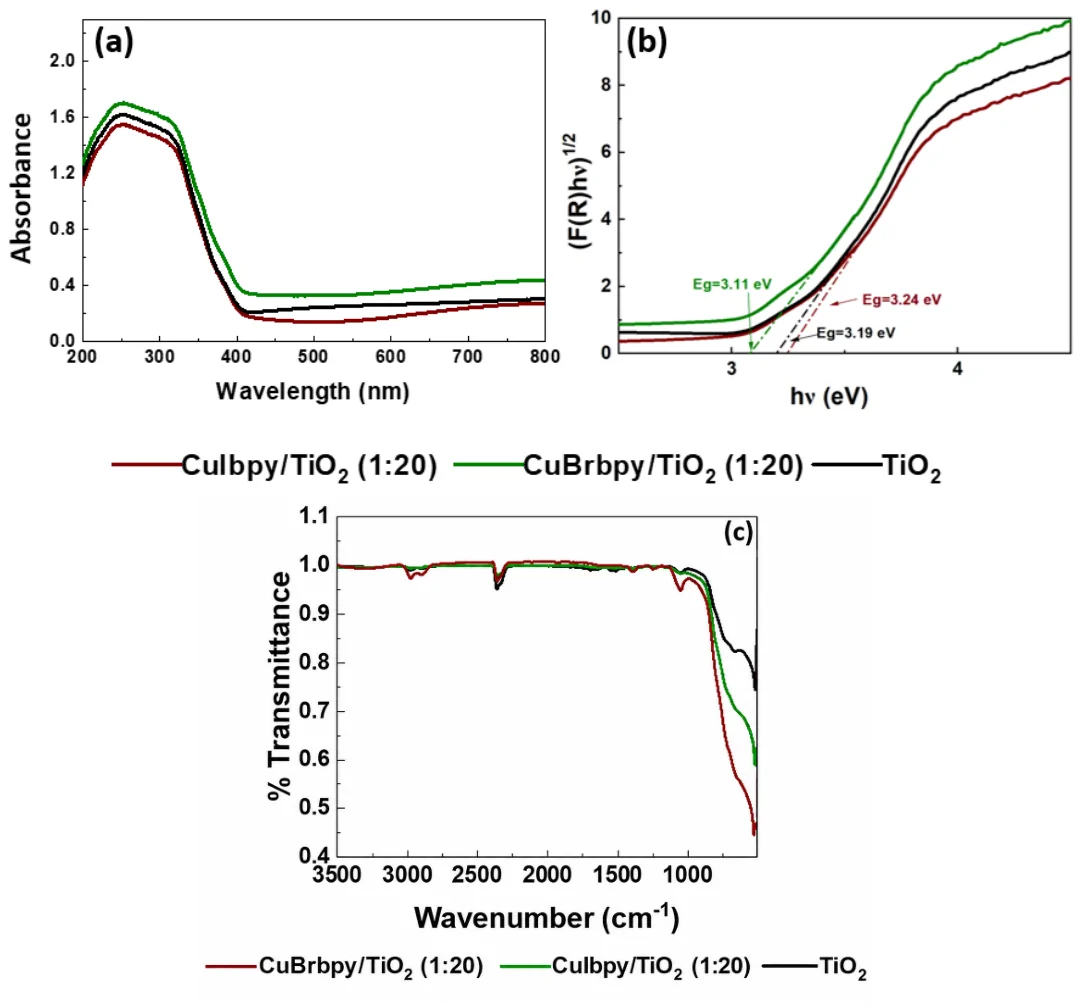
UV–visible DRS spectra of TiO_2_, CuXbpy/TiO_2_ (1:20 mass ratio) (X = I or Br) (**a**) Kubelka–Munk plots for indirect band gap energies TiO_2_, CuXbpy/TiO_2_ (1:20 mass ratio) (X = I or Br) (**b**) and FTIR spectra for TiO_2_, CuXbpy/TiO_2_ (1:20 mass ratio) (X = I or Br) (**c**).

The optical properties of TiO_2_-P25 and its composite with CuIbpy and CuBrbpy MOFs were studied by DRS (Figure 3a). TiO_2_ exhibits a prominent absorption band in the range of 200–400 nm mainly due to electronic transitions from the valence band (consisting of oxygen 2p orbitals) to the conduction band (consists of titanium 3d and 4s orbitals) corresponding to the referenced band gap transition of TiO_2_ [46]. Compared to pristine TiO_2,_ the CuXbpy/TiO_2_ (1:20 mass ratio) (X = I or Br) composite materials exhibit absorption throughout the entire UV-visible light spectrum, attesting to a good integration of CuXbpy (X = I or Br) and TiO_2_-P25 in the composite. The origin of the absorption in the visible is metal-to-ligand charge transfer in CuXbpy (X = I or Br) MOFs. The CuBrbpy/TiO_2_ composite exhibits a prominent absorption band below 400 nm, attributed to the presence of TiO_2_, but the absorption edge appears slightly red-shifted compared to bare TiO_2_. This observation suggests that in the CuBrbpy/TiO_2_ (1:20 mass ratio) composite, the electronic band structure of TiO_2_ is influenced by its interaction with CuBrbpy. The band gap of the CuBrbpy-TiO_2_ (1:20 mass ratio) composite is slightly smaller (3.11 eV) than that of bare TiO_2_ (3.19 eV), as determined by Kubelka–Munk plots (Figure 3b). In the case of CuIbpy/TiO_2_ (1:20 mass ratio) composite, a very slight blue shift is observed in its absorption edge compared to bare titania, which results in a very small increase of the apparent optical band gap (3.24 eV for the composite compared with 3.19 eV for bare TiO_2_) (Figure 3b). This can indicate that a higher energy is required for electron excitation from the valence band (VB) to the conduction band (CB). The Burstein–Moss effect could be one phenomenon that could explain this slight shift [47,48].

The Burstein–Moss effect is a well-known phenomenon in which the lowest energy states of the conduction band of an n-type semiconductor become partially filled with electrons, which are close in energy, from additional elements in the lattice or in the composite. This filling raises the Fermi level, increasing the apparent optical band gap and, thus, the energy needed for electron excitation from the VB to the CB.

In the case of CuIbpy/TiO_2_, the empty 3d and 4s orbitals of titanium forming the CB of TiO_2_ are likely filled by electrons from the iodide 5p orbitals, which are expected to lie slightly higher in energy than the CB of TiO_2_, resulting in an observed increase in the apparent optical band gap. In contrast, this effect is not observed in the CuBrbpy composite. This absence may be attributed to differences in the electronic configuration of bromine ([Ar]3d^10^ 4s^2^ 4p^5^), which places its highest occupied level sufficiently lower in energy than the CB of TiO_2_, preventing the filling of the conduction band [49]. To confirm this effect, photoluminescence, Mott–Schottky and Hall measurements are still required and will be performed in our future study. We also carried out XRD measurements to check whether this small change in the band gap comes from a structural modification of TiO_2_. The diffraction patterns of TiO_2_-P25 and of CuIbpy/TiO_2_ (1:20) are superimposable (Figure S6, Supporting Information), showing the characteristic reflections of the anatase and rutile phases of P25 with no shift in peak position and no change in peak width or relative intensity after deposition of the MOF. The crystalline structure of TiO_2_ is therefore preserved in the composite, and the slight variation of the apparent band gap is not related to a structural change of the semiconductor. The peaks of CuIbpy are not visible in the pattern of the composite; this is due the low MOF content (0.8 wt% Cu).

#### 3.2.2. FTIR Analysis

FTIR spectra were recorded to study the interactions between CuXbpy (X = I or Br) and TiO_2_. The spectra are represented in Figure 3c and reveal characteristic bands related to the presence of CuXbpy and TiO_2_ in the CuXbpy/TiO_2_ (X = I or Br) composite materials. However, the intensity of these bands is low because of the small CuXbpy/TiO_2_ mass ratio (1:20 mass ratio). The broad absorption below 1000 cm^−1^ in the composite indicates significant changes and shifts compared to bare TiO_2_. This behavior has been associated with Ti-O-C vibrations, indicating that Ti-O-C bonds may play an important role in the coupling between TiO_2_ and carbon atoms of the ligand [50,51].

#### 3.2.3. Morphology Characterization

**Figure 4:**
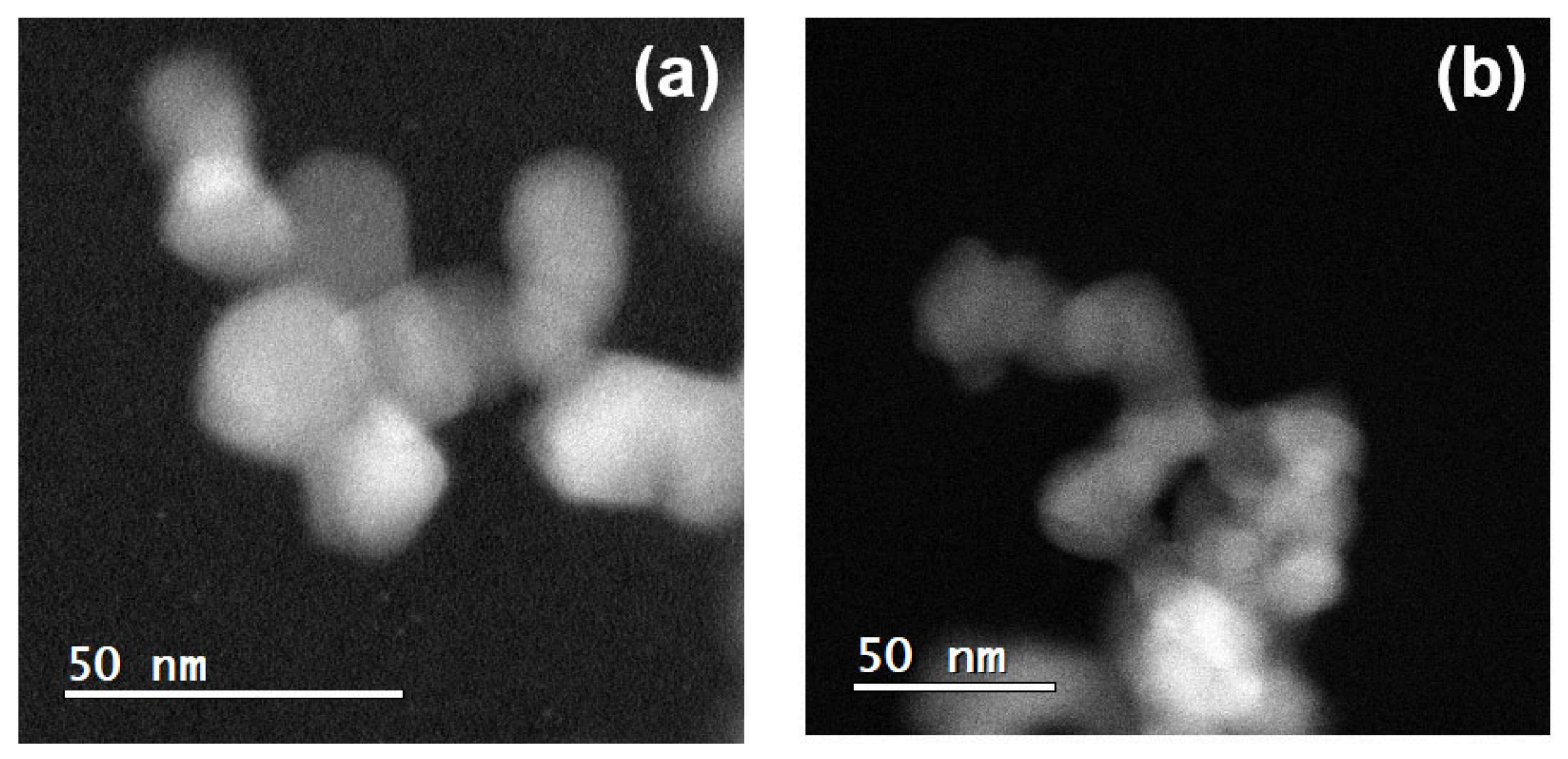
HAADF-STEM images of the CuIbpy/TiO_2_ (1:20 mass ratio) (**a**) and CuBrbpy/TiO_2_ (1:20 mass ratio) composites (**b**).

HAADF-STEM images of the pristine CuXbpy (X = I or Br) materials showed no evidence of distinct Cu-based nanoparticles or large Cu aggregates, indicating that the Cu species remain embedded within the coordination framework (Figure S7, Supporting Information). Following the composite formation with TiO_2_, well-defined crystalline TiO_2_ nanoparticles were clearly seen; however, no segregated Cu-based nanophases were observed (Figure 4). This observation is consistent with the low copper loading in the composites, for a CuXbpy/TiO_2_ mass ratio (1:20 mass ratio), to the copper loadings of 0.8 wt% Cu in CuIbpy/TiO_2_ and 1.0 wt% Cu in CuBrbpy/TiO_2_. The absence of segregated Cu phases points to a homogeneous distribution of the CuXbpy (X = I or Br) MOFs component across the TiO_2_ surface, ensuring intimate interfacial contact between the two materials. However, the presence of very small copper clusters below the detection limit cannot be ruled out here.

#### 3.2.4. XPS Analysis for the CuBrbpy, CuXbpy/TiO_2_ (1:20 Mass Ratio) (X = I or Br)

The survey scan of pristine CuBrbpy MOF was conducted over two areas (Figure S8a, Supporting Information), indicating the presence of copper (Cu), carbon (C), bromine (Br), nitrogen (N), and oxygen (O) species in the material. The survey scan for both areas is exactly the same, indicating that the elements are homogeneously distributed. Furthermore, Figure S8b–e (Supporting Information) illustrates the fitted core-level spectra for Cu 2p, Br 3d, C 1s, and N 1s. For the Cu 2p core-level spectrum, Figure S7b confirms the presence of copper species, mainly as Cu(I) species, as indicated by the absence of satellite peaks, with Cu 2p_3/2_ peak at ~932 eV and its Cu 2p_1/2_ peak at ~952 eV. The Br 3d core level spectrum is fitted with two components (Figure S7c) at binding energies of 68 eV (3d_5/2_ spin orbital) and 69 eV (3d_3/2_ spin orbital), further confirming the presence of bromine in the MOF structure. For C 1s core level spectrum (Figure S7d), the peaks located at binding energies (BE) of 284.6 eV, 285.4 eV, and 286.8 eV correspond to C=C, C=N (present in the bipyridine structure), and C-O (that might be due to interactions involving adventitious carbon contamination), respectively. For the N 1s core level spectrum (Figure S8e, Supporting Information), the peak centered at a binding energy of 399.4 eV was attributed to the N=C group bounded with copper present in the bipyridine structure.

**Figure 5:**
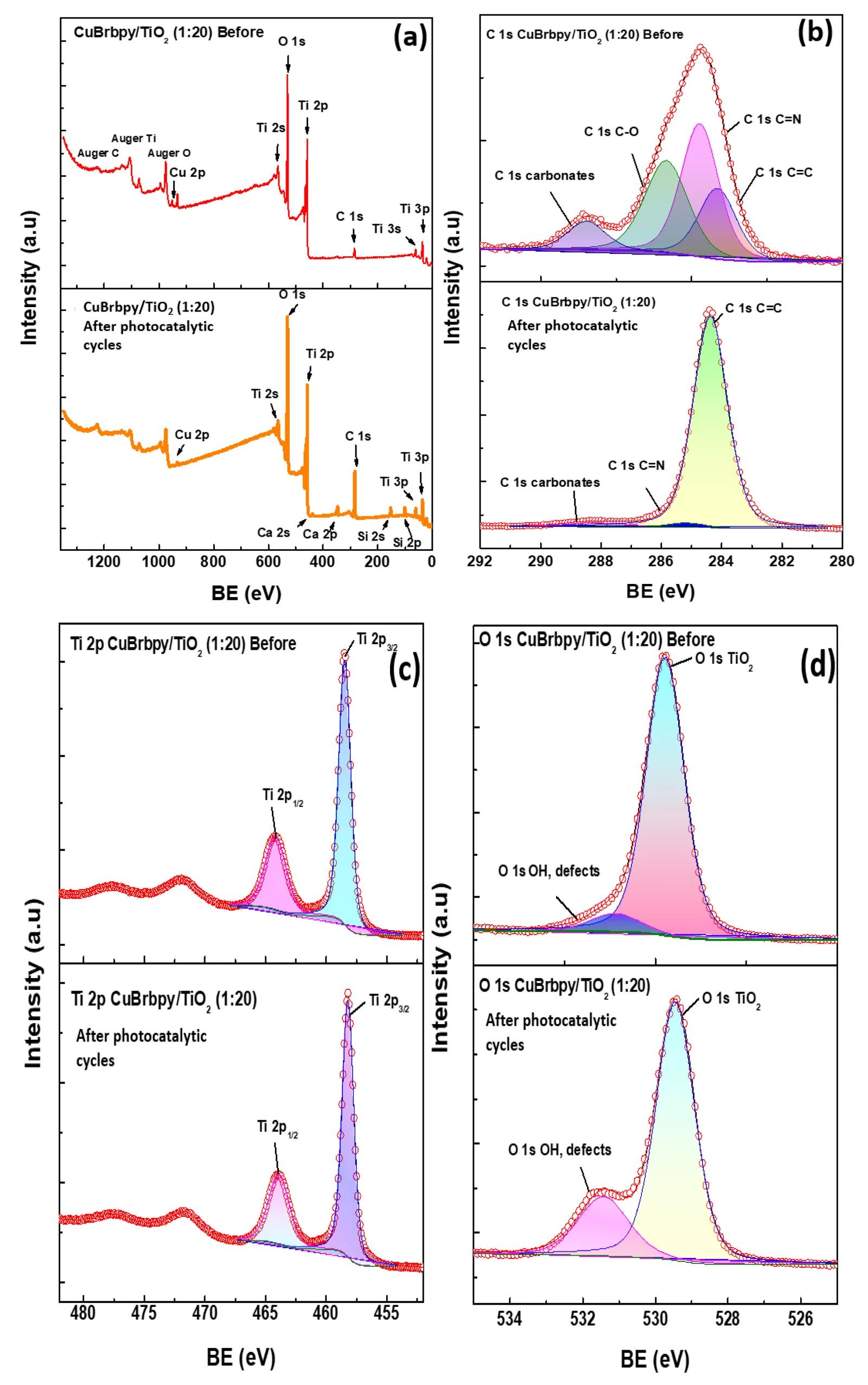
XPS survey spectrum (**a**) and core level spectra: C 1s (**b**) Ti 2p (**c**) O 1s (**d**) for the CuBrbpy/TiO_2_ (1:20 mass ratio) MOF composite material, before and after four photocatalytic cycles. In (**b**–**d**), the red open circles represent the experimental data, the solid lines passing through the red circles represent the overall fitted envelope, the shaded areas represent the individual deconvoluted components, and the flat line along the baseline represents the background. For N 1s core level spectra before photocatalysis (Figure 6a), it can be observed that all the nitrogen atoms are in the same chemical environment as in pristine CuBrbpy MOF (Figure S7e), coordinated to copper, with only one contribution deconvoluted from the spectrum at a BE of 399.6 eV. Finally, for Cu 2p core level spectra (Figure 6b), Cu is present in a +1-oxidation state before photocatalysis.

The core level spectra of C 1s, Ti 2p, and O 1s for CuIbpy/TiO_2_ (1:20 mass ratio) before and after four photocatalytic cycles are represented in Figure 5a–d.

In the survey scan of CuBrbpy/TiO_2_ (1:20 mass ratio) before photocatalysis (Figure 5a), the presence of Cu, Ti, C, and O elements is observed. However, bromide is not clearly detected, indicating that it is either not efficiently bonded to the composite or leached out during the centrifugation phase of the synthesis process.

The C 1s core level spectra were fitted with four peaks (Figure 5b), C=N (the most prominent, indicative of bpy coordination in the MOF), followed by C-O, C=C, and carbonates at BE of 284.6 eV, 285.8 eV, 284.1 eV, and 288.4 eV, respectively. The ratios Cu:N for CuBrbpy and CuBrbpy/TiO_2_ before vary roughly from 1:1 to 2:1; however, it is never 1:2, as expected. There is an excess of Cu, which increases when TiO_2_ is present. The Ti 2p core level spectra are deconvoluted into two distinct spin-orbit components (Figure 5c) attributed to Ti 2p_3/2_ and 2p_1/2_ at binding energy (BE) 458.5 eV and 464.2 eV, respectively.

However, after four photocatalytic cycles (16 h of use), a survey scan of CuBrbpy/TiO_2_ (1:20 mass ratio) composite revealed that copper was barely detectable (Figure 5a), suggesting significant leaching and instability in the composite. Additionally, the presence of silica and calcium can be detected after photocatalysis. This may result from the vials, sample handling, or contaminants from the environment. For C 1s core level spectra, after photocatalytic cycles, the intensity of C=N is significantly reduced (compared to before photocatalysis cycles) (Figure 5b), suggesting that the C=N bond is breaking due to the degradation of the framework. The shape of Ti 2p core level spectra shows no changes before and after photocatalysis cycles (Figure 5c), indicating that titanium is stable throughout the photocatalytic process.

**Figure 6:**
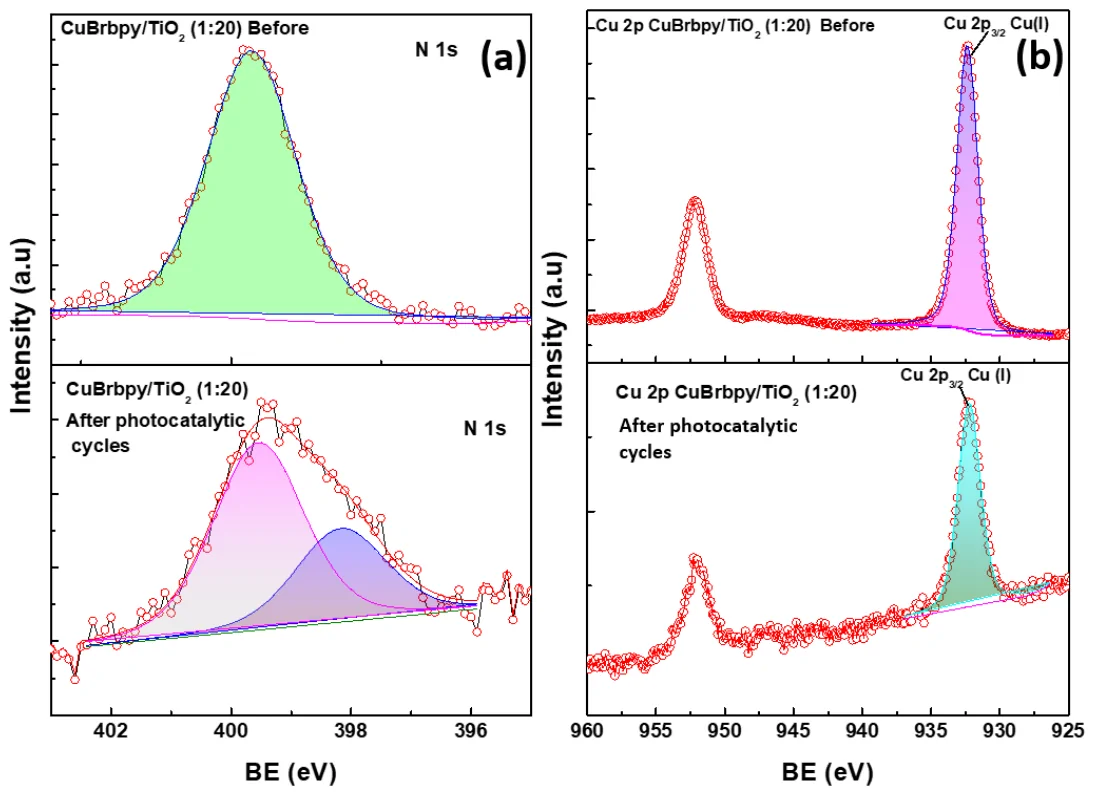
XPS core level spectra: N1s (**a**) and Cu 2p (**b**) for the composite CuBrbpy/TiO_2_ (1:20 mass ratio) before and after four photocatalytic cycles. Red open circles represent the experimental data, the solid lines passing through the red circles represent the overall fitted envelope, the shaded areas represent the individual deconvoluted components, and the flat line along the baseline represents the background.

For N 1s core level spectra, after photocatalysis cycles (Figure 6a), the N 1s signal is weak, and the signal-to-noise ratio is low, suggesting that there are only a few percentages of nitrogen left in the sample, which is likely due to leaching. A similar observation was made from the Cu 2p signal. Additionally, in the N 1s spectrum after photocatalysis cycles, two contributions were considered: the first one at 399.5 eV, corresponding to the preserved composite, and another peak at 398.1 eV, indicating changes due to degradation of the photocatalyst. Finally, for Cu 2p core level spectra (Figure 6b) after four photocatalytic cycles (Figure 6b), copper is detected in very low quantities, most probably because of leaching indicating the non-stability of the composite with cycling.

The trend for core level spectra of C 1s, Ti 2p, O 1s, and Cu 2p for CuIbpy/TiO_2_ (1:20 mass ratio) before and after four photocatalytic cycles (Figure S8, Supporting Information) is similar to that observed in CuBrbpy/TiO_2_ (1:20 mass ratio) composite material before and after photocatalytic cycling.

Finally, the XPS results point a possible modification of the initial MOF structure in contact to TiO_2_ accompanied by the loss of Br (for XPS, about a few nm probing depth) at the surface, and a change in the Cu:N ratio. In addition, FTIR results indicate the formation of Ti-O-C bonds.

#### 3.2.5. Charge Carriers’ Dynamics Studied by TRMC

**Figure 7:**
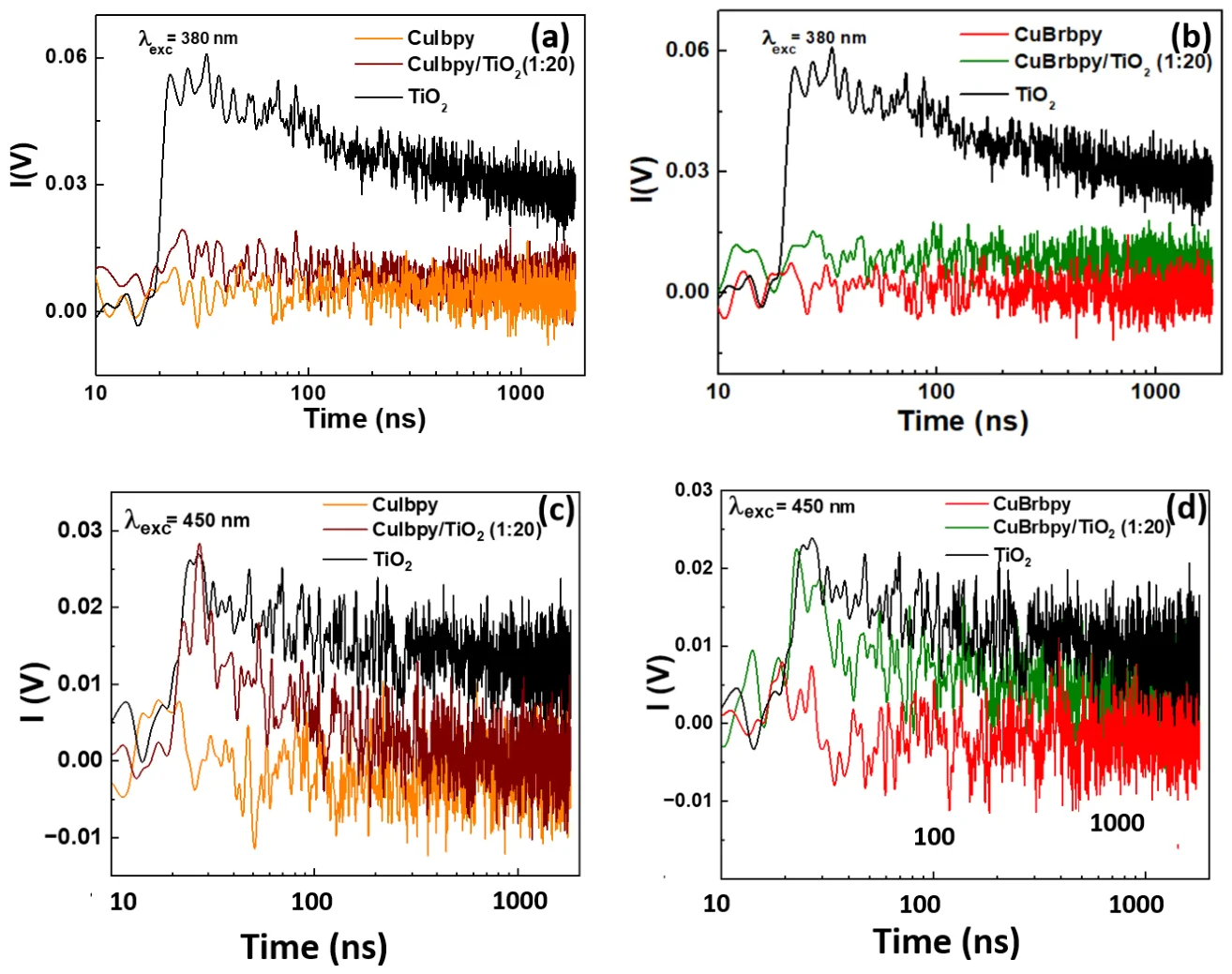
Logarithmic TRMC signals plot of pristine CuXbpy (X = I or Br), TiO_2_ and the composites CuXbpy/TiO_2_ (1:20 mass ratio) (X = I or Br) with a λ_exc_ = 380 nm (**a**,**b**) and 450 nm (**c**,**d**) and laser intensity of 0.5 mJ and 4.3 mJ, respectively.

TRMC signals of TiO_2_-P25, bare MOFs (CuBrbpy and CuIbpy), and their composites CuXbpy/TiO_2_ (1:20 mass ratio) (X = I or Br) composites, after excitation at 380 and 450 nm, are shown in Figure 7. The signals are related to electrons in the conduction band after laser excitation at 380 nm (Figure 7a,b). However, for CuXbpy/TiO_2_ composites, the signal intensity decreases considerably. This decrease can be attributed to three effects: (1) because of surface MOF, TiO_2_ receives less illumination, (2) the surface MOF acts as a defect site, increasing surface recombination, (3) photogenerated electrons are transferred from the CB of TiO_2_ to CuXbpy.

Under visible excitation (Figure 7c,d), TiO_2_ exhibits a weak signal, and the signal is decreased compared to the signal observed in the UV range because TiO_2_ is inactive in the visible range. However, for CuXbpy/TiO_2_ (1:20 mass ratio) (X = Br, I) composites, a small signal at 450 nm appears, and the intensity of the TiO_2_ signal decreases. This observation can be explained by an electron transfer in the opposite direction; it means the ejection of electrons from CuXbpy to the CB of TiO_2_. This signal, in the visible range, indicates that this composite may be active in this spectral region.

#### 3.2.6. Photoelectrochemical Studies

**Figure 8:**
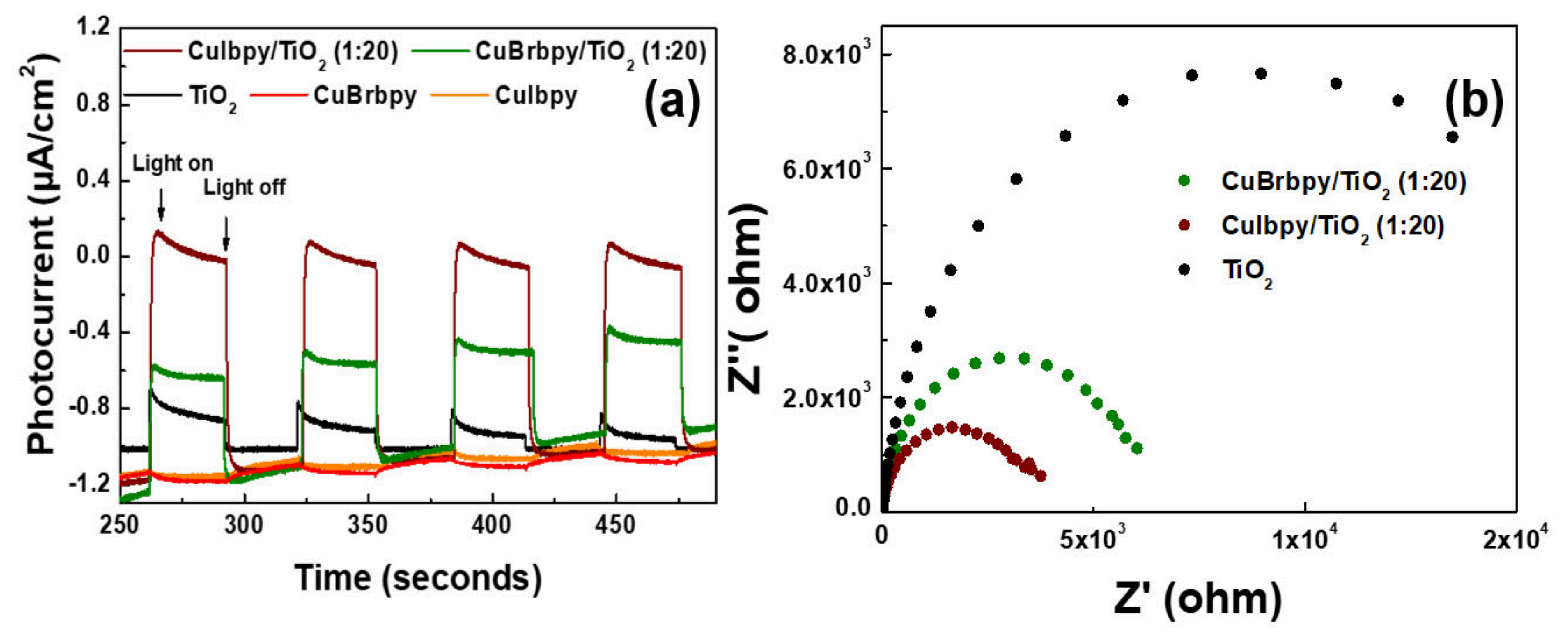
Photocurrent transient responses at a constant potential of 0 V on fluorine thin oxide (FTO) electrodes for several on/off cycles of intermittent irradiation 30 s for CuXbpy, CuXbpy/TiO_2_ (1:20 mass ratio) (X = I or Br), and TiO_2_ (**a**), and Nyquist plots of electrochemical Impedance spectroscopy (EIS) curve of TiO_2_ and the composite CuXbpy/TiO_2_ (X = I or Br) (1:20 mass ratio) (**b**) Experimental conditions: 0.5 M Na_2_SO_4_ (electrolyte), pH = 7.2.

The behavior of photogenerated charge carriers in the samples TiO_2_, pristine CuIbpy MOFs, pristine CuBrbpy MOF, CuIbpy/TiO_2_ (1:20 mass ratio) composite, and CuBrbpy/TiO_2_ (1:20 mass ratio) composite, was also studied by transient photocurrent (Figure 8a).

An immediate spike was observed when the light was turned on, indicating the instant generation of charge carriers in all the samples. After turning off the light, the signal subsides, indicating charge carrier recombinations. The composite CuIbpy/TiO_2_ (1:20 mass ratio) shows the highest current compared to CuBrbpy/TiO_2_ (1:20 mass ratio), TiO_2,_ and pristine CuXbpy (X = I or Br) MOFs. This may indicate more efficient charge carrier separation in CuIbpy/TiO_2_ (1:20 mass ratio). Pristine CuXbpy (X = I or Br) MOFs exhibit very low photocurrent, indicating that the junction with TiO_2_ is essential to boost the activity of the composite material. Furthermore, the decay observed during illumination revealed that the decay for CuXbpy/TiO_2_ (1:20 mass ratio) (X = I or Br) was less steep, indicating better separation of charge carriers than bare TiO_2_.

Furthermore, electrochemical impedance spectroscopy (EIS) was used to examine the charge transfer behavior and internal resistance of the electrode materials. In the Nyquist plots (Figure 8b), the semi-circle observed in the high frequency region corresponds to the interfacial charge transfer resistance (R_CT_), with a smaller diameter indicating lower resistance between the interfaces and more efficient charge transport across the interfaces. As shown in the Figure 8b, bare TiO_2_ exhibits the largest semi-circle, suggesting the highest charge transfer resistance compared to the composite materials. After coupling TiO_2_ with CuXbpy (X = I or Br) MOFs, the semicircle diameter decreases significantly, showing improved charge-transfer properties. Among the composite materials, CuIbpy/TiO_2_ (1:20 mass ratio) shows a smaller semi-circle compared to the one of CuBrbpy/TiO_2_ (1:20 mass ratio) indicating that CuIbpy/TiO_2_ has a small interfacial charge transfer resistance, which facilitates more efficient separation of charge carriers, thereby enhancing the overall photocatalytic activity. Therefore, both EIS and photocurrent measurements indicate better charge carrier separation with CuIbpy/TiO_2_ composite compared to pristine CuBrbpy MOF material.

#### 3.2.7. Photocatalytic Tests

**Figure 9:**
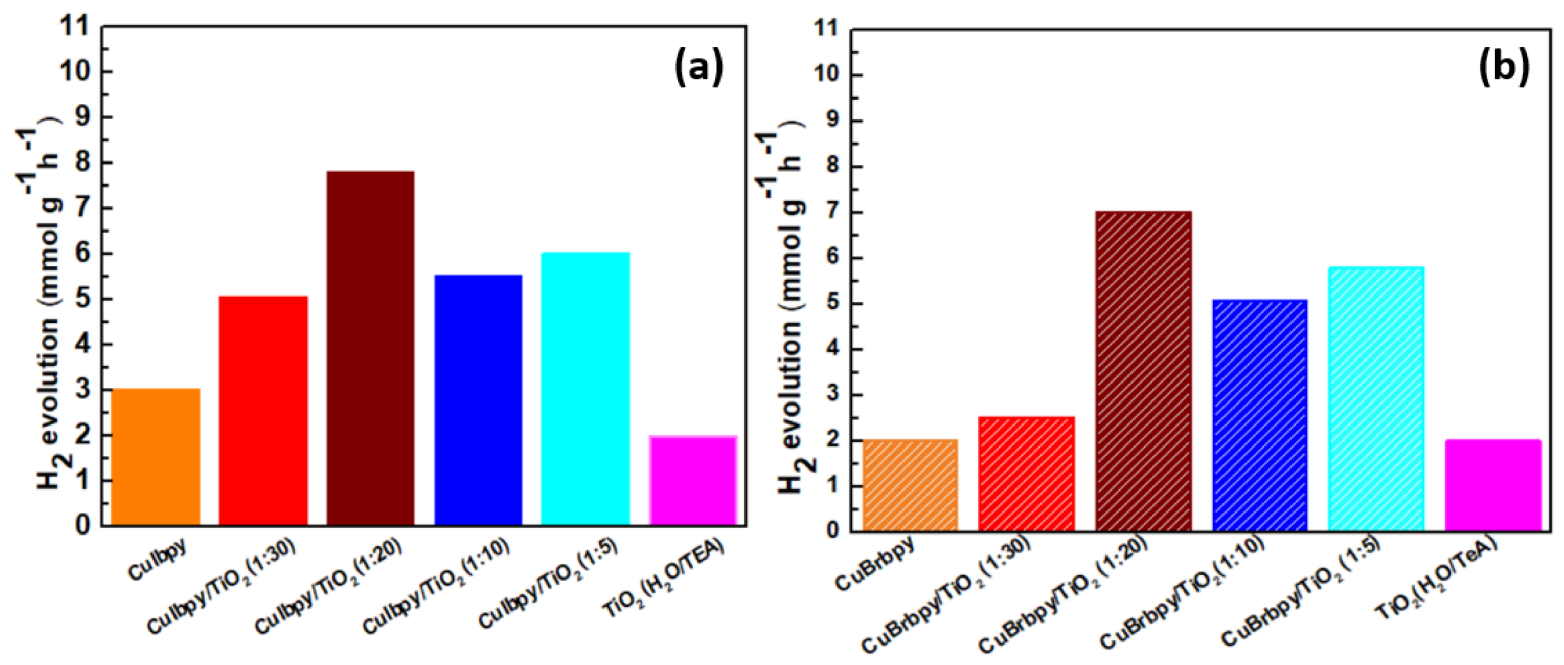
Photocatalytic hydrogen generation for TiO_2_, CuXbpy, and the CuXbpy/TiO_2_ (X = I or Br) composite with different mass ratios; (**a**) Hydrogen production with CuXbpy/TiO_2_ (1:20 mass ratio) (X = I or Br) after four photocatalytic cycles; (**b**) Experimental conditions: 6.66 mg of the sample dispersed in 20 mL of the V_TEA_/V_water_ = 1/19, V_reactor_ = 37 mL reactor, Hg lamp 500 W, 4 h radiation per run.

Firstly, the photocatalytic activity of pristine materials was evaluated. CuBrbpy, CuIbpy and TiO_2_ materials reached a hydrogen production rate of 2.0 mmol g^−1^ h^−1^, 3.0 mmol g^−1^ h^−1^ and 1.9 mmol g^−1^ h^−1^, respectively. A control experiment was carried out under identical conditions in the absence of photocatalyst that gave 2.1 mmol g^−1^ h^−1^, normalized to the 6.66 mg of catalyst used in all other experiments, indicating that TEA undergoes direct photolysis under UV irradiation. The mass ratio of CuXbpy (X = I or Br) to TiO_2_ of the composite was then optimized for photocatalytic hydrogen generation. The experiments were conducted by using various mass ratios (1:5), (1:10), (1:20), (1:30) (Figure 9a,b) of the composites CuIbpy/TiO_2_ and CuBrbpy/TiO_2_. The 1:20 mass ratio was found to be the most active in terms of photocatalytic hydrogen generation with a production rate of 7.8 mmol g^−1^ h^−1^ and 7.0 mmol g^−1^ h^−1^ for the CuIbpy/TiO_2_ and CuBrbpy/TiO_2_ composites. The composites showed much higher photocatalytic activity than bare TiO_2_ and pristine CuXbpy (X = I or Br) MOFs. This is consistent with the photocurrent and impedance results, which showed better charge carrier separation and lower internal resistance with the composite (compared with the bare components). These results are also competitive or even better than the performances of the reported reference composite materials of the literature listed in Table S1.

It has to be noted that the color of the CuXbpy/TiO_2_ (1:20 mass ratio) (X = I or Br) composites changes immediately upon irradiation, transitioning from white to grey, as shown in Figure S9 (Supporting Information). This color change was reversible as the white color was restored after overnight exposure to air. TRMC signals show that electrons are trapped by the MOF after irradiation of the composite. The color change of the composite after irradiation also suggests reduction of copper; this is also reversible, with Cu being reoxidized under O_2_. This reversible behavior was already observed and documented in the case of HKUST/TiO_2_ [12,13], where the change in color was attributed to reduction of copper and formation of small copper clusters. A similar phenomenon of Cu reduction is already known for Cu-exchanged zeolite materials [52]. The reversible change in color of the composite corresponds to the reduction and oxidation of Cu species.

The reusability and the stability of the photocatalysts are important factors for application in photocatalysis. The hydrogen production (in mmol/g) of the two composite is represented as a function of time (in hours), for four photocatalytic cycles, in Figure S10 (Supporting Information).

A decrease in hydrogen production was observed after the two first photocatalytic cycles (Figure S10, Supporting Information) before stabilization at 1.9 and 1.7 mmol g^−1^ h^−1^ for CuIbpy/TiO_2_ and CuBrbpy/TiO_2_, respectively (averaged over cycles 3 and 4). For comparison, the CuO (TiO_2_) (1.5 wt% Cu) reference catalyst synthesized by radiolysis on titania delivered 2.2 mmol g^−1^ h^−1^ [13]. The stabilized activity of the composites is thus of the same order as that of a dispersed copper oxide phase on TiO_2_, which suggests that the remaining activity after the third cycle could originate from a new stable phase (e.g., Cu or Cu oxides nanoparticles on TiO_2_). After comparing the XPS spectra of the composites before and after photocatalysis, it was revealed that the primary reason is the leaching of copper and nitrogen, accompanied by degradation of the bipyridine framework, which progressively induces a decrease in the photocatalytic hydrogen activity. In addition, catalyst particles can aggregate, reducing specific surface area and hence decreasing photocatalytic hydrogen production. Finally, structural degradation of the catalyst due to prolonged exposure to light could also affect the photocatalytic activity for hydrogen generation.

The structural evolution of the composites after cycling could not be characterized by XRD. In our previous work on HKUST-1/TiO_2_ composites, the MOF diffraction peaks of were not detectable at mass ratios below 1:3 because of the low MOF content [13]. The copper content of the CuIbpy/TiO_2_ and CuBrbpy/TiO_2_ composites is even lower (0.8–1.0 wt%), and any Cu or Cu-oxide phase formed during cycling would likely be diluted and too small to give detectable signals. These results can be further compared with our previous work on HKUST-1/TiO_2_ (1:20 mass ratio) [13], which exhibited 5.1 mmol g^−1^ h^−1^ of H_2_ during the first photocatalytic cycle with a higher Cu loading of 1.5 wt%. Here again, the composites CuXbpy/TiO_2_ (X = I or Br) exhibit much higher activity than the pristine CuXbpy (X = I or Br) MOFs. CuIbpy/TiO_2_ (with only 0.8 wt% Cu loading) and CuBrbpy/TiO_2_ (with only 1.0 wt% Cu loading) delivered 7.8 mmol g^−1^ h^−1^ and 7.0 mmol g^−1^ h^−1^ of H_2_, respectively. When normalized to copper content, this corresponds to 8.9 and 7.0 mmol g^−1^ h^−1^ per wt% Cu for CuIbpy/TiO_2_ and CuBrbpy/TiO_2_, respectively, against 3.2 mmol g^−1^ h^−1^ per wt% Cu for HKUST-1/TiO_2_. Therefore, the CuIbpy/TiO_2_ and CuBrbpy/TiO_2_ composites exhibit roughly 2.8 and 2.2 times higher catalytic efficiency per copper unit than HKUST-1/TiO_2_, respectively. Furthermore, the catalytic activity for H_2_ evolution of CuIbpy/TiO_2_ is comparable to that of 1 wt% Pt (TiO_2_) (7.0 mmol g^−1^ h^−1^), measured in the same reactor (37 mL) and under the same irradiation (500 W Hg lamp), using 1 wt% Pt (TiO_2_) prepared by the same radiolytic method as in our previous work [13]. The two experiments differ in the sacrificial electron donor (methanol for 1 wt% Pt (TiO_2_), triethylamine for the composites), and since triethylamine is generally a more efficient hole scavenger than methanol, the two values should be regarded as comparable rather than indicating superior performance of the composites. These results suggest that Cu(I)-based bipyridine architectures may serve as effective noble-metal-free alternatives even at low metal loadings.

The photocatalytic stability with cycling of the two Cu-MOF/TiO_2_ systems differs markedly: HKUST-1/TiO_2_ becomes progressively more active, reaching 13.2 mmol g^−1^ h^−1^ after six photocatalytic cycles and thereby surpassing 1 wt% Pt(TiO_2_). ICP-OES and XPS confirmed no significant copper leaching; the gain in activity was attributed to reversible copper redox transformations between Cu^2+^, Cu^+^, and Cu^0^, and stabilization of the reduced copper species by the trimesic acid ligand [12,13]. In contrast, the CuXbpy/TiO_2_ (X = I or Br) composites lose activity across successive runs, with copper and nitrogen leaching detected by XPS. However, despite a gradual decrease of photocatalytic activity across successive cycles, the CuXbpy/TiO_2_ (X = I or Br) composites exhibit excellent initial performance. During the first cycle, hydrogen production rates of 7.8 and 7.0 mmol g^−1^ h^−1^ were obtained for CuIbpy/TiO_2_ (1:20 mass ratio) and CuBrbpy/TiO_2_ (1:20 mass ratio), respectively. These rates are clearly competitive with previously reported noble-metal-free pristine MOF photocatalysts [18]. The higher photocatalytic activity of the CuXbpy/TiO_2_ (X = I or Br) composites compared to that of bare MOF materials is mainly due to more effective charge carrier separation and enhanced light harvesting. The composites also outperform when compared to other low-loading Cu/TiO_2_ materials such as compounds containing 0.5 wt% Cu by impregnation, 1.3 wt% CuO by impregnation, and 1.2 wt% Cu by incipient wetness, which typically deliver between 1.0 and 3.2 mmol g^−1^ h^−1^ (Table S1) [53,54,55,56,57,58,59,60,61,62].

## 4. Proposed Photocatalytic Mechanism

**Figure 10:**
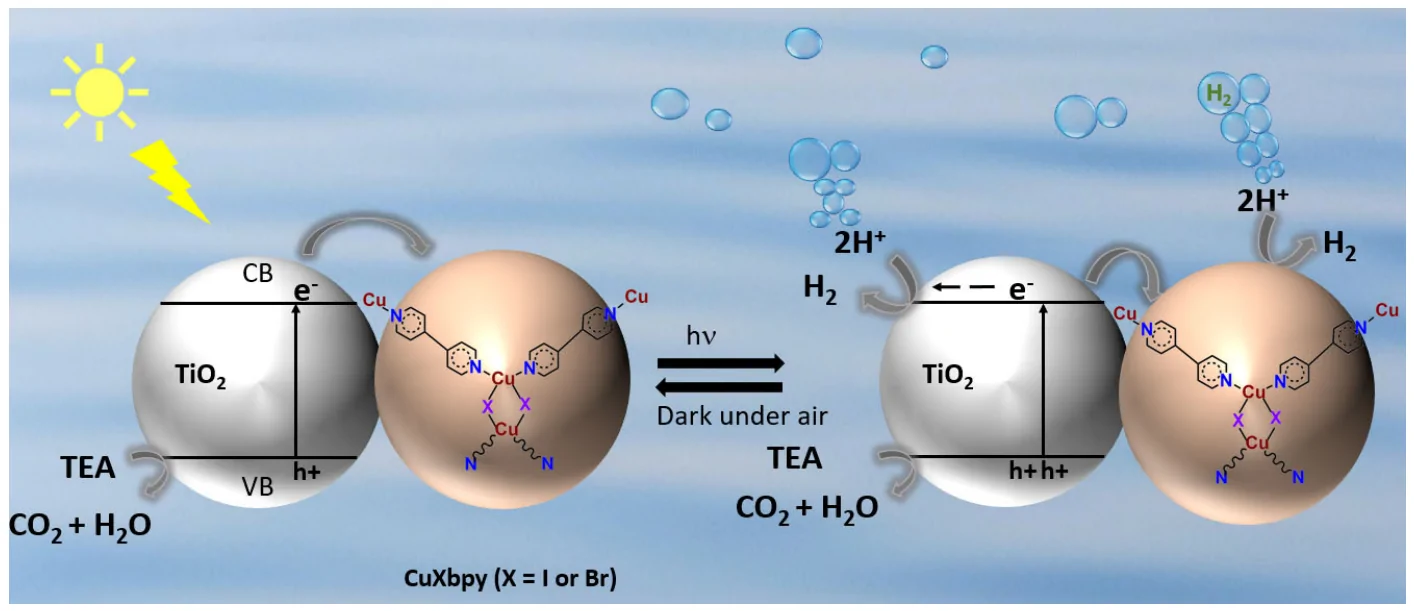
Proposed mechanism for hydrogen generation with the composite HKUST-1/TiO_2_ (1:20 mass ratio) under UV-light excitation.

The results presented above demonstrate that the photocatalytic activity of TiO_2_ is significantly enhanced after association with CuXbpy (X = I or Br), resulting in the formation of CuXbpy (X = I or Br)/TiO_2_ composites materials. The improved photocatalytic activity results from heterojunction formation, which could facilitate a synergetic effect of the two constituents. A proposed mechanism which can explain how the synergy of CuXbpy (X = I or Br) MOFs and TiO_2_ may inhibit electron-hole recombinations is represented in Figure 10. Moreover, understanding the mechanism could also be beneficial in order to design a more stable photocatalyst.

Under UV irradiation, photogenerated electrons of TiO_2_ are injected into its CB, and they are subsequently scavenged by the CuMOF, as evidenced by the TRMC (Figure 7) signals. Meanwhile, the photogenerated holes are consumed by TEA, which acts as a sacrificial reagent. The enhanced separation of photogenerated charge carriers is supported by increased transient photocurrent responses, reduced charge-transfer resistance (observed in electrochemical impedance spectroscopy), and lower TRMC signal intensities of the composite materials compared to bare TiO_2_. The color change of the composite after irradiation also suggests the reduction of copper, which is also reversible, with Cu being reoxidized under O_2_ [12,13].

Previous DFT studies performed on HKUST-1/TiO_2_ composites showed that ligand environment surrounding the copper centers plays a crucial role in determining the structural and electronic properties of the framework, including Cu-Cu distances, charge distribution, and the tendency of Cu species to undergo structural reorganization [13]. The studies highlighted the significant role of ligand chemistry in governing the charge transfer processes at the MOF/TiO_2_ interface and ultimately affecting the overall photocatalytic behavior of Cu-based MOF/TiO_2_ composite systems.

In the present work, the Cu(I) centers are coordinated by bipyridine ligands rather than carboxylate linkers, resulting in a unique coordination environment around the copper ions. There are no observable Cu nanoparticles or Cu nanoclusters in the TEM analysis of the as-synthesized CuXbpy/TiO_2_ (X = I or Br) composites materials, unlike HKUST-1/TiO_2_ composite material, although very small copper clusters below the detection limit of the technique cannot be excluded. This could explain the leaching of copper and nitrogen confirmed by XPS analysis. It also suggests that the enhanced photocatalytic activity is mainly attributed to enhanced charge separation and interfacial charge transfer across the CuXbpy/TiO_2_ (X = I or Br) composite materials heterojunction. This conclusion is further supported by enhanced photocurrent response, reduced charge-transfer resistance, and improved charge-carrier separation.

## 5. Conclusions

In conclusion, here again Cu-based MOFs composites show very high activity for photocatalytic hydrogen generation, this activity being much higher than that of the pristine materials. The CuBrbpy/TiO_2_ (1:20 mass ratio) and CuIbpy/TiO_2_ (1:20 mass ratio) composite materials exhibit high photocatalytic hydrogen generation of 7.8 and 7.0 mmol g^−1^ h^−1^, respectively. These hydrogen production rates are close to the performance of 1 wt% Pt (TiO_2_) benchmark (7.0 mmol g^−1^ h^−1^) and outperforms HKUST-1/TiO_2_ (1:20 mass ratio) composite material in its first cycle (5.1 mmol g^−1^ h^−1^), using less than 1 wt% copper and no noble metal. This enhanced photocatalytic activity is mainly due to interfacial charge transfer at the CuXbpy/TiO_2_ (X = I or Br) composite materials heterojunction, as supported by photocurrent, EIS, and TRMC measurements. However, contrary to HKUST-1/TiO_2_ material, these composites showed limited stability with cycling, probably due to the leaching of copper and nitrogen during photocatalysis, as evidenced by XPS. These results highlight the importance of simultaneously tailoring the electronic structure and the coordination framework stability. Further, Operando XPS, X-ray absorption spectroscopy (XAS) and EXAFS experiments, complemented with in situ EPR, will be performed together with theoretical calculations to provide an in-depth insight into the underlying mechanism and to understand the active sides. Future developments should therefore concentrate on developing ligand architectures that not only promote the charge transfer, but also provide enhanced stabilization of copper species during photocatalytic operation.

## Data Availability

The original contributions presented in this study are included in the article/Supplementary Materials. Further inquiries can be directed to the corresponding authors.

## Supplementary Materials

The following supporting information can be downloaded at: https://www.mdpi.com/article/10.3390/nano16191232/s1, Figure S1: Schematic presentation of CuIbpy and CuBrbpy MOFs synthesis. In CuX, X represents I or Br, ACN stands for acetonitrile, and bpy is the 4,4′-bipyridine ligand.; Figure S2: Quartz reactor and its corresponding dimensions; Figure S3: Image of the Mercury lamp used (a), with its respective radiation spectrum (b); Figure S4: TGA graph illustrating the decomposition of Cu-I-bpy (a,c); and Cu-Br-bpy (b,d) before and after activation, respectively; Figure S5: Simulated XRD pattern based on crystallographic data from CCDC 118797-118801, comparing the 2θ positions with the experimental data of the CuIbpy and CuBrbpy MOFs; Figure S6: TEM Images of bare CuIbpy (a) CuBrbpy (b) MOF materials; Figure S7: XPS survey spectrum (a) and core level spectra: Cu 2p (b) Br 3d (c) C 1s (d) N 1s (e) for the pristine CuBrbpy MOF material; Figure S8: XPS survey spectra (a) and core level spectra of: C 1s (b) Ti 2p (c) O 1s (d) N1s (e) and (f) Cu 2p for the CuIbpy/TiO_2_ (1:20 mass ratio) MOF composite material before and after four photocatalytic cycles; Figure S9: Image illustrating the color difference of composite CuXbpy/TiO_2_ (1:20 mass ratio) (X = I or Br) dispersed (1 mg/mL) in water (containing TEA) in quartz reactors after 4 h of irradiation in 500 W Mercury (Hg) lamp and after back oxidation in air (after 1 h); Figure S10: Hydrogen production with CuXbpy/TiO_2_ (1:20 mass ratio) (X = I or Br) after 4 photocatalytic cycles; Experimental conditions: 6.66 mg of the sample dispersed in 20 mL of the VTEA/Vwater = 1/19, Vreactor = 37 mL reactor, Hg lamp 500 W, 4 h radiation per run; Table S1: Comparison of photocatalytic hydrogen generation rates for typical composite materials with the present study.
