# Supplementary material for: CuXBpy-MOF/TiO2 (X = I or Br) Composite Nanomaterials for Photocatalytic Hydrogen Generation
Source: Nanomaterials (Basel). 2026 Sep 30;16(19):1232. doi: 10.3390/nano16191232 (PMC13666697; doi:10.3390/nano16191232)
Supplement: Supplementary file 1 [file nanomaterials-16-01232-s001.zip › nanomaterials-4515429-supplementary.pdf]

Supplementary Materials

$CuXBpy$-MOF/TiO_2 (X = I or Br) Composite Nanomaterials for Photocatalytic Hydrogen Generation

Alisha Khan [1], Marie Le Pivert [1,2], Hiba EL Idrissi Bouyahyaoui [1], Md Faiz Alam [3], Diana Dragoe [4], Daniel Bahena Uribe [5], Alireza Ranjbari [1], Christophe Colbeau-Justin [1], Johnny Deschamps [3,*] and Hynd Remita [1,*]

1 Institut de Chimie Physique (ICP), UMR 8000 CNRS, Université Paris-Saclay, 91405 Orsay, France
2 Laboratoire de Réactivité de Surface (LRS), UMR CNRS 7197, Sorbonne Université, 75005 Paris, France
3 Unité Chimie et Procédés (UCP), ENSTA Paris, Institut Polytechnique de Paris, 91120 Palaiseau, France
4 Institut de Chimie Moléculaire et des Matériaux d'Orsay (ICMMO), UMR 8182 CNRS, Université Paris-Saclay, 91405 Orsay, France
5 Laboratorio Avanzado de Nanoscopía Electrónica (LANE), Centro de Investigación y de Estudios Avanzados del Instituto Politécnico Nacional (CINVESTAV), 07360 Mexico City, Mexico
* Correspondence: johnny.deschamps@ensta.fr (J.D.); hynd.remita@universite-paris-saclay.fr (H.R.)

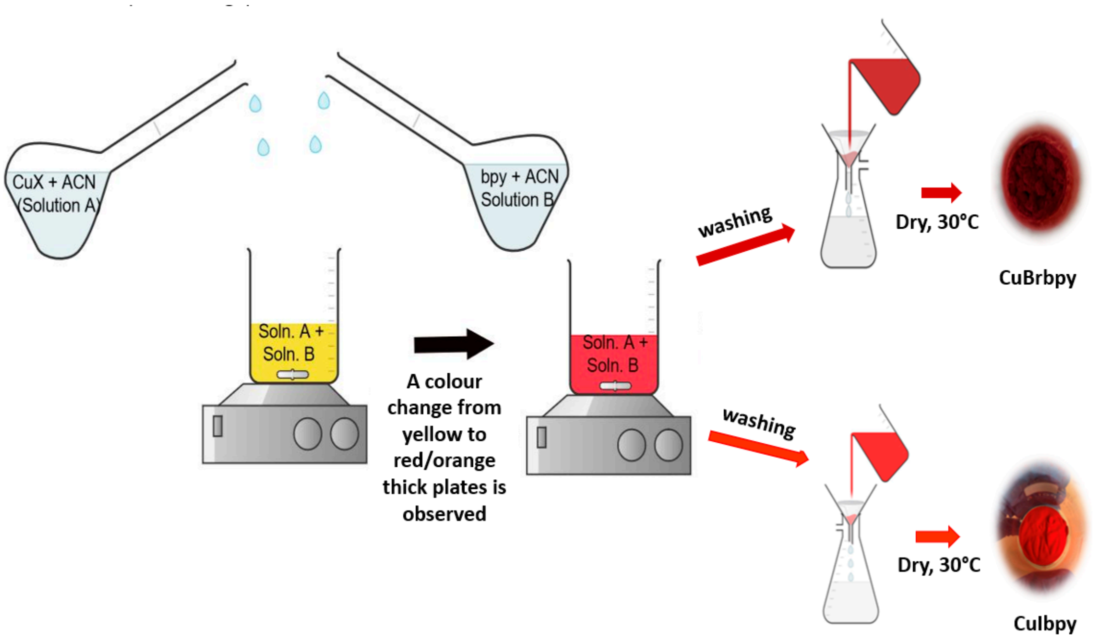

Figure S1. Schematic presentation of CuIbpy and CuBrbpy MOFs synthesis. In CuX, X represents I or Br, ACN stands for acetonitrile, and bpy is the 4,4′-bipyridine ligand.

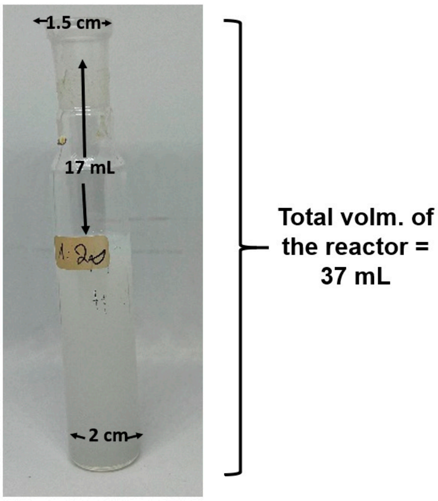

Figure S2. Quartz reactor and its corresponding dimensions.

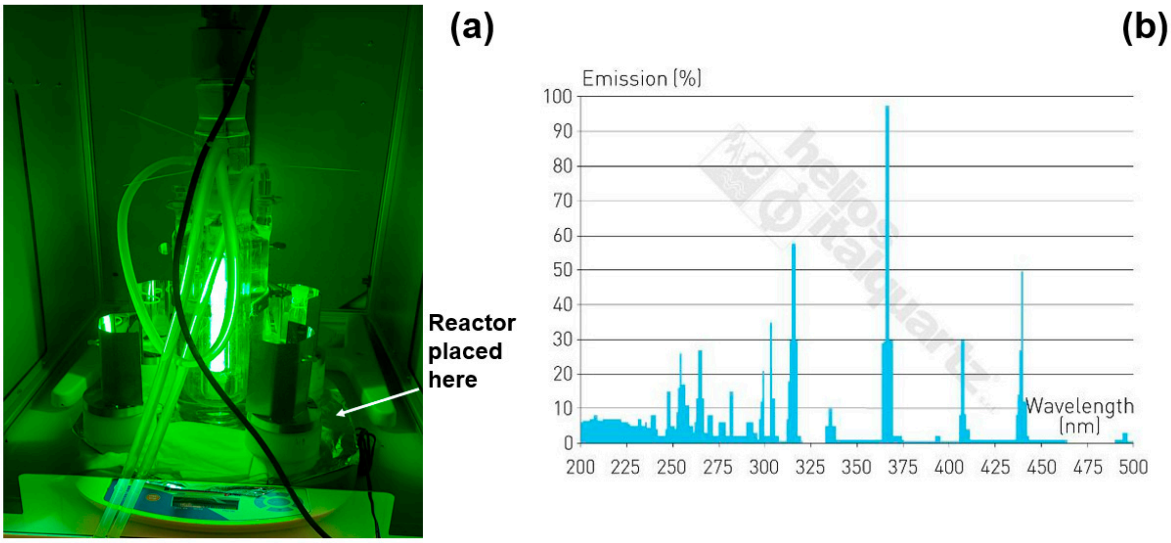

Figure S3. Image of the Mercury lamp used (**a**), with its respective radiation spectrum (**b**).

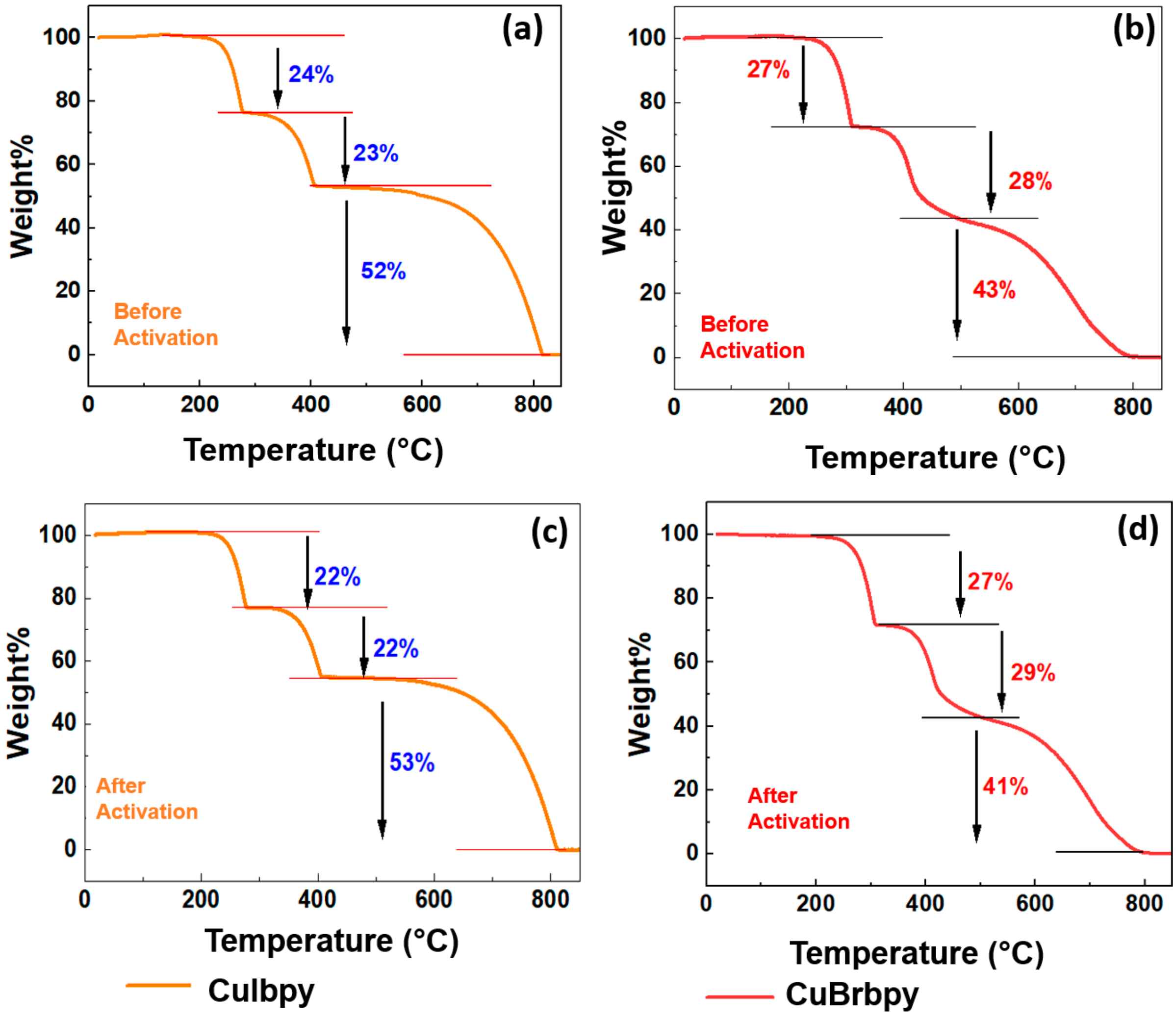

Figure S4. TGA graph illustrating the decomposition of Cu-I-bpy **(a,c)**; and Cu-Br-bpy **(b,d)** before and after activation, respectively.

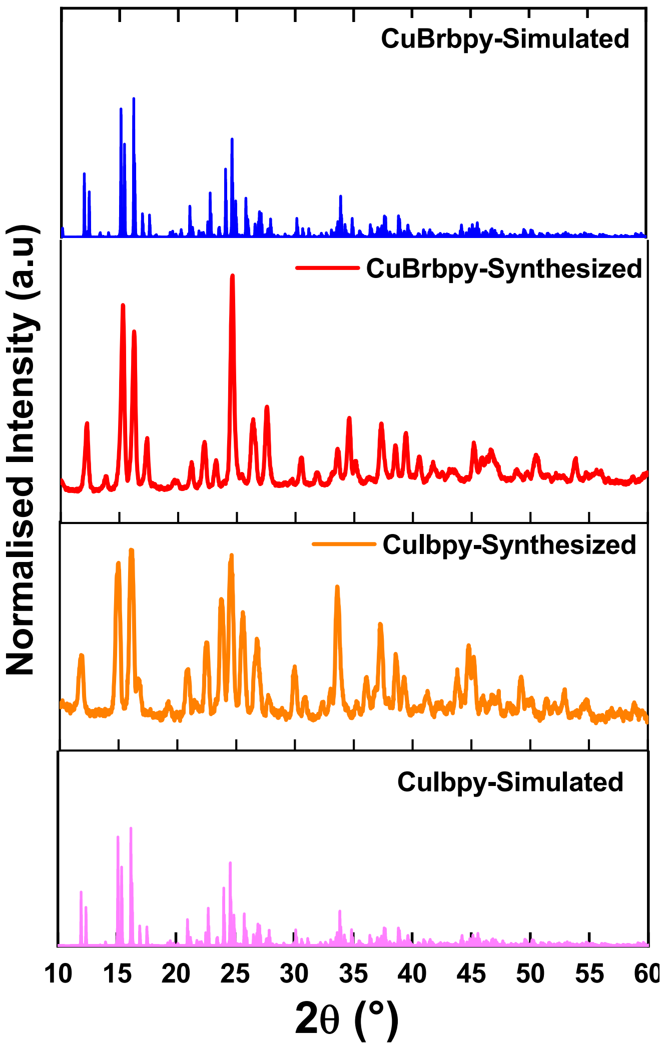

Figure S5. Simulated XRD pattern based on crystallographic data from CCDC 118797-118801, comparing the 2θ positions with the experimental data of the CuIbpy and CuBrbpy MOFs.

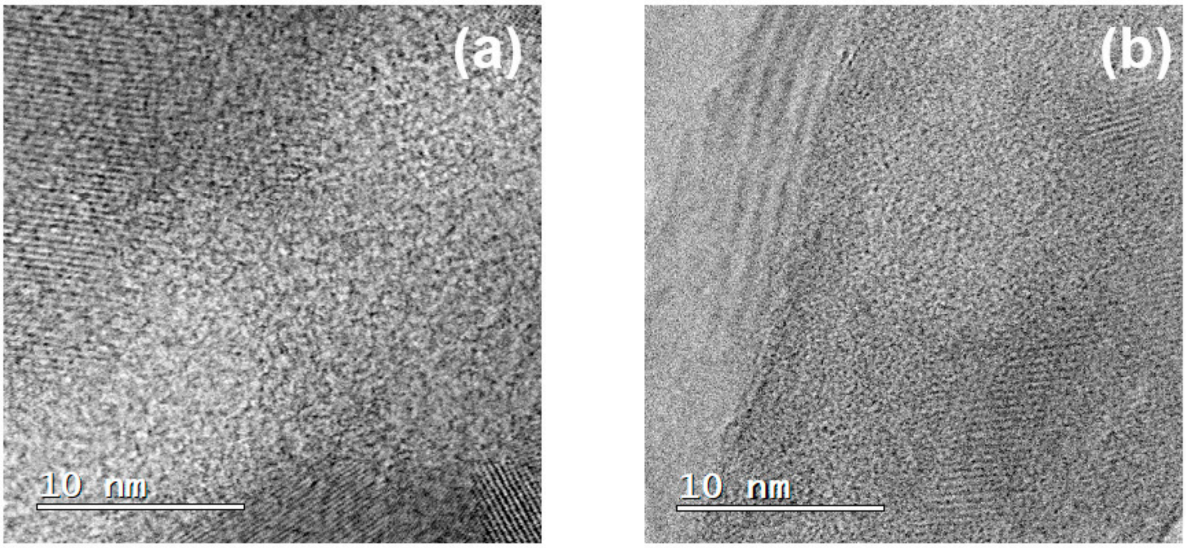

Figure S6. TEM Images of bare CuIbpy (**a**) CuBrbpy (**b**) MOF materials.

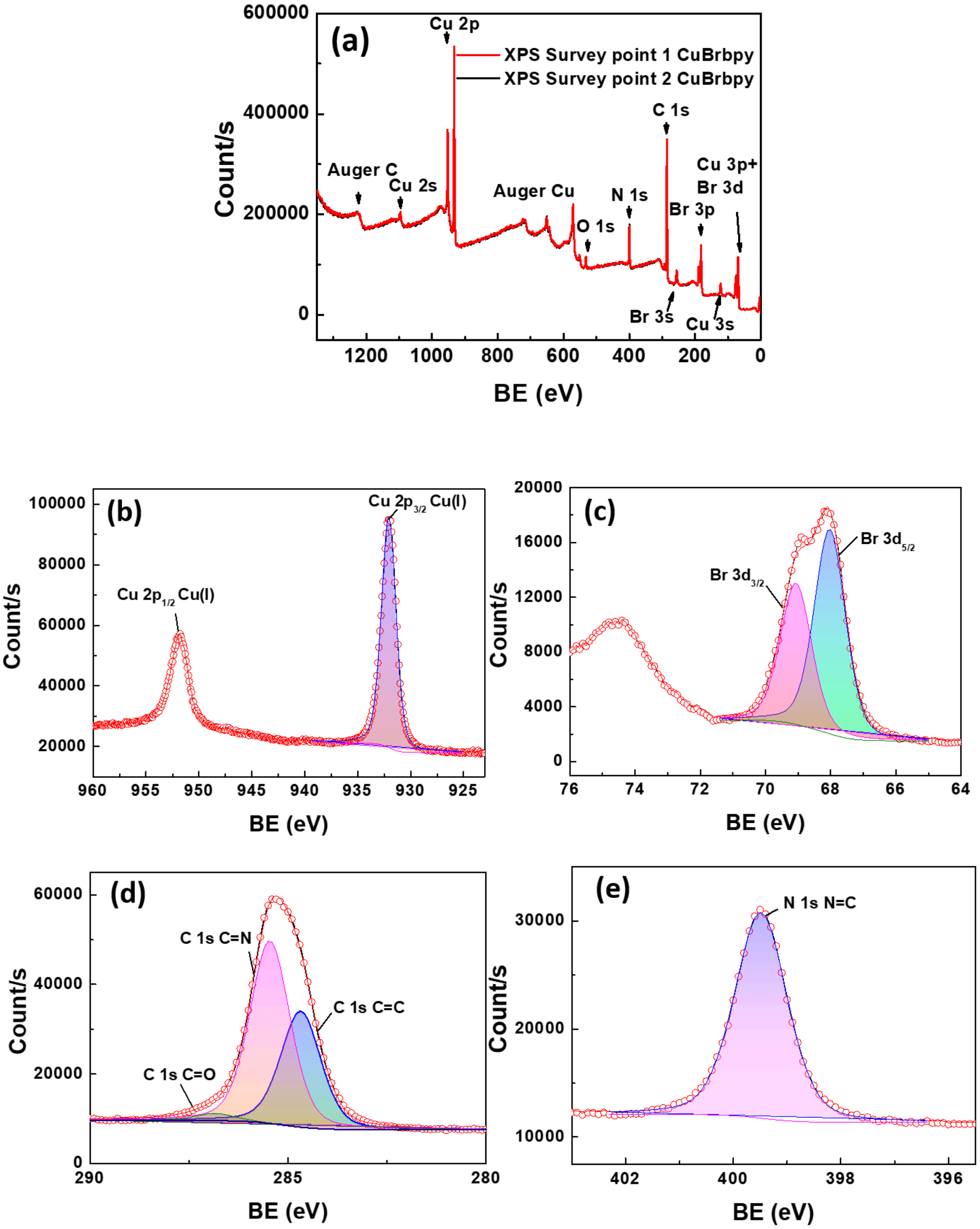

Figure S7. XPS survey spectrum (**a**) and core level spectra: Cu 2p (**b**) Br 3d (**c**) C 1s (**d**) N 1s (**e**) for the pristine CuBrbpy MOF material.

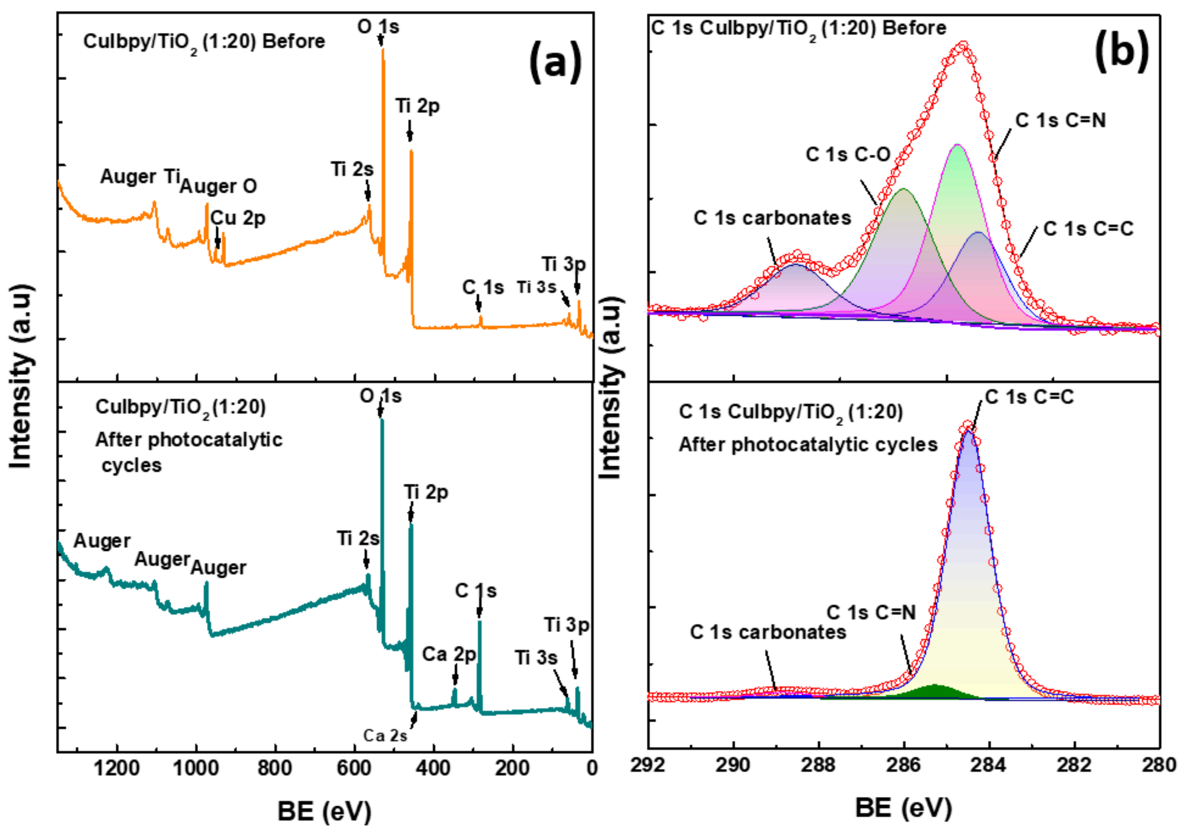

(a)
CuIbpy/TiO2 (1:20) Before
O 1s
Ti 2p
Auger Ti
Auger O
Cu 2p
Ti 2s
C 1s
Ti 3s
Ti 3p
CuIbpy/TiO2 (1:20)
After photocatalytic cycles
Auger
Auger
Auger
Ca 2p
Ca 2s
Intensity (a.u)
BE (eV)
(b)
C 1s CuIbpy/TiO2 (1:20) Before
C 1s C=N
C 1s C-O
C 1s carbonates
C 1s C=C
C 1s CuIbpy/TiO2 (1:20)
After photocatalytic cycles
Intensity (a.u)
BE (eV)

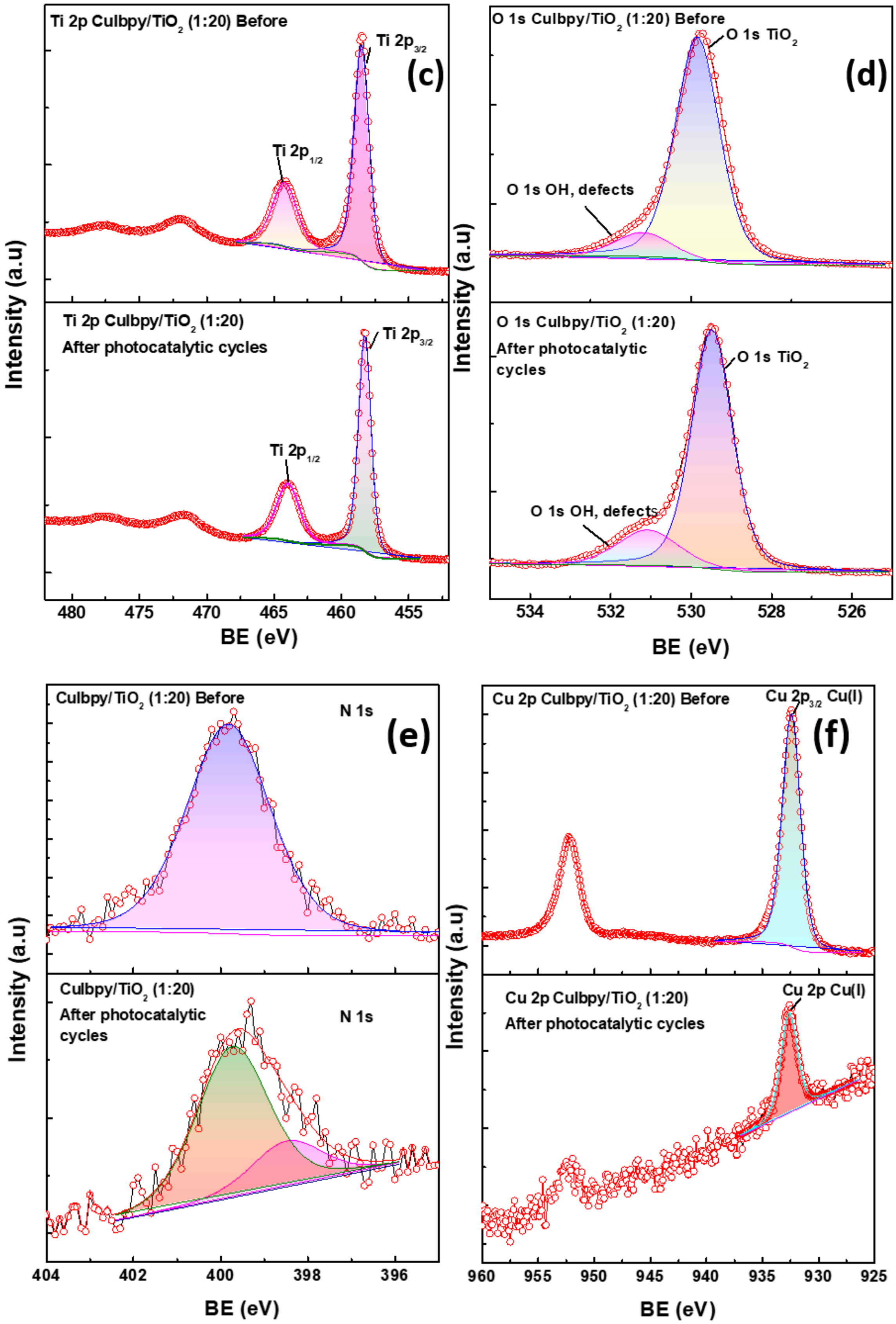

Figure S8. XPS survey spectra (**a**) and core level spectra of: C 1s (**b**) Ti 2p (**c**) O 1s (**d**) N1s (**e**) and (**f**) Cu 2p for the CuIbpy/TiO_2 (1:20 mass ratio) MOF composite material before and after four photocatalytic cycles.

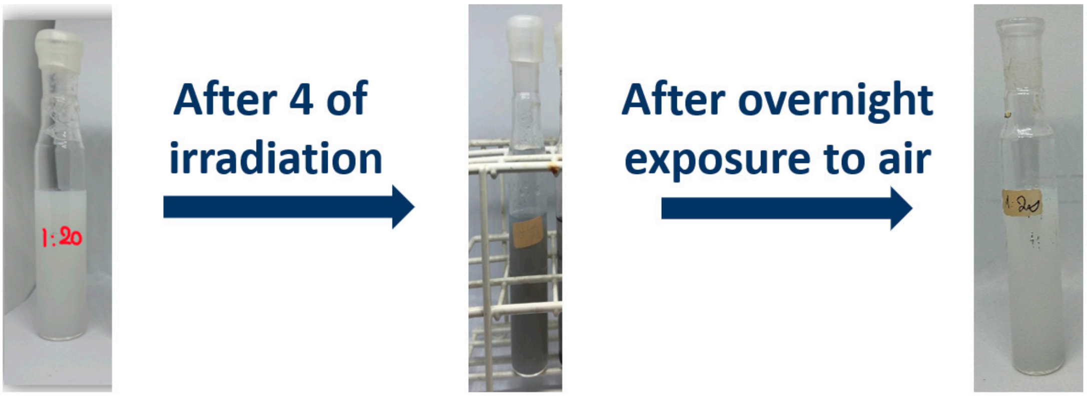

Figure S9. Image illustrating the color difference of composite CuXbpy/TiO_2 (1:20 mass ratio) (X = I or Br) dispersed (1 mg/mL) in water (containing TEA) in quartz reactors after 4 hours of irradiation in 500 W Mercury (Hg) lamp and after back oxidation in air (after 1 h).

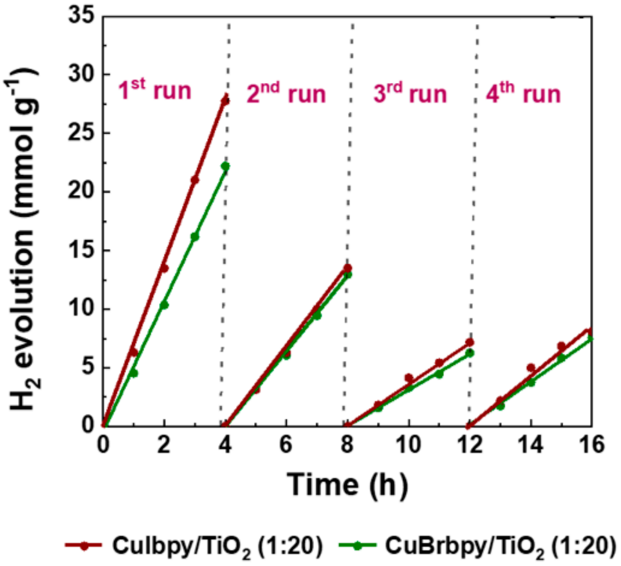

Figure S10. Hydrogen production with CuXbpy/TiO_2 (1:20 mass ratio) (X = I or Br) after 4 photocatalytic cycles; Experimental conditions: 6.66 mg of the sample dispersed in 20 mL of the V_{TEA}/V_{water} = 1/19, Vreactor = 37 mL reactor, Hg lamp 500 W, 4 h radiation per run

Table S1. Comparison of Photocatalytic Hydrogen generation rates for typical composite materials with the present study

**Photocatalyst**	**Sacrificial reagents (vol%)**	**Light source**	**H_2 evolution (mmol g^{-1} h^{-1})**	**Ref.**
**CuIbpy/TiO_2 (mass ratio 1:20) (0.87 wt% Cu)**	5 % TEA-H_2O	500 W Hg lamp	7.8	Present study
**CyIbpy/TiO_2 (mass ratio 1:20) (1 wt% Cu)**	5 % TEA-H_2O	500 W Hg lamp	7.0	Present study
**HKUST-1/TiO_2 (mass ratio 1:20) (1.5 wt% Cu)**	25% MeOH-H_2O	300 W Xe lamp	5.1	[1]
**HKUST-1/TiO_2 (mass ratio 1:20) (1.5 wt% Cu) after six photocatalytic tests (After R6)**	25% MeOH-H_2O	300 W Xe lamp	13.2	[1]
**UiO-66(COOH)$_2$-Cu**	-	Visible + NIR	6.1	[2]
**CuI-bpy**	RhB pollutant	300 W Xe lamp (λ > 420 nm)	0.02	[3]
**CeO_2/CuI-bpy**	RhB pollutant	300 W Xe lamp (λ > 420 nm)	0.15	[3]
**TiO_2/HKUST-1 (mass ratio 1:1)**	1:9 vol. ratio Gly-H_2O	150 solar simulator	4.5	[4]
**29 mol% $Cu(OH)_2$/P25 (precipitation)**	0.09 M EG-H_2O	3 W LEDs (UV)	3.4	[5]
**1.2 wt% Cu/TiO_2-anatase (incipient wetness)**	30% MeOH-H2O	208 W (UV) lights	3.2	[6]
**1.3 wt% CuO/P25 (impregnation)**	0.1 M Gly-H_2O	3 W LEDs (UV)	2.0	[7]
**3.0 wt% CuO/P25 (impregnation)**	25% MeOH-H_2O	300 W Xe lamp	4.1	[1]
**Ni-Pd/TiO_2**	MeOH(1/1)	400 W Hg arc	4.2	[8]
**0.5 wt% Cu/P25 (impregnation)**	10% MeOH-H_2O	300 W Xe lamp	1.1	[9]
**10 wt% Cu/TiO_2 (complex precipitation)**	6% MeOH-H_2O	500 W Hg lamp	0.40	[10]
**2wt% Pt-TiO_2**	MeOH(1/4)	350 W Xe lamp	16.7	[11]
**CoOx/TiO_2/Pt**	MeOH (1/5)	………….	7.9	[12]
**Cu-NH2-MIL 125 (Ti)**	ACN:TEA:H_2O (63:15:2)	32 W >420 nm	0.40	[13]

M = Molar; H_2O = Water; ACN = Acetonitrile, TEA = Triethylamine, MeOH = Methanol; EtOH = Ethanol; Gly = Glycerol; EG = Ethelene Glycol

References

1. Khan, A.; Le Pivert, M.; Ranjbari, A.; Dragoe, D.; Bahena-Uribe, D.; Colbeau-Justin, C.; Herrero, C.; Rutkowska-Zbik, D.; Deschamps, J.; Remita, H. Cu-Based MOF/TiO_2 Composite Nanomaterials for Photocatalytic Hydrogen Generation and the Role of Copper. *Adv. Funct. Mater. n/a*, 2501736, doi:10.1002/adfm.202501736.
2. Abou Khalil, Z.; Nath, A.; Hannouche, K.; Assaad, N.; Mouchaham, G.; Beauvois, A.; Briois, V.; Serre, C.; Hmadeh, M.; Daturi, M.; et al. Dual-Photon–Driven Hydrogen Evolution in Copper-Based Photocatalysts under near-Infrared and Visible Light. *Nat. Commun.* **2025**, *17*, 308, doi:10.1038/s41467-025-67014-x.
3. Bu, D.; Yang, C.; Bu, D.; Huang, S. Co-Catalyst Free Direct Z–Scheme Photocatalytic System with Simultaneous Hydrogen Evolution and Degradation of Organic Pollutants. *Int. J. Hydrog. Energy* **2023**, *48*, 576–585, doi:10.1016/j.ijhydene.2022.09.196.
4. Martínez, F.M.; Albiter, E.; Alfaro, S.; Luna, A.L.; Colbeau-Justin, C.; Barrera-Andrade, J.M.; Remita, H.; Valenzuela, M.A. Hydrogen Production from Glycerol Photoreforming on TiO_2/HKUST-1 Composites: Effect of Preparation Method. *Catalysts* **2019**, *9*, 338, doi:10.3390/catal9040338.
5. Yu, J.; Ran, J. Facile Preparation and Enhanced Photocatalytic H2-Production Activity of $Cu(OH)_2$ Cluster Modified TiO2. *Energy Environ. Sci.* **2011**, *4*, 1364–1371, doi:10.1039/C0EE00729C.
6. Wu, N.-L.; Lee, M.-S. Enhanced TiO_2 Photocatalysis by Cu in Hydrogen Production from Aqueous Methanol Solution. *Int. J. Hydrog. Energy* **2004**, *29*, 1601–1605, doi:10.1016/j.ijhydene.2004.02.013.
7. Yu, J.; Hai, Y.; Jaroniec, M. Photocatalytic Hydrogen Production over CuO-Modified Titania. *J. Colloid Interface Sci.* **2011**, *357*, 223–228, doi: 10.1016/j.jcis.2011.01.101.
8. Luna, A.L.; Dragoe, D.; Wang, K.; Beaunier, P.; Kowalska, E.; Ohtani, B.; Bahena Uribe, D.; Valenzuela, M.A.; Remita, H.; Colbeau-Justin, C. Photocatalytic Hydrogen Evolution Using Ni-Pd/TiO_2: Correlation of Light Absorption, Charge-Carrier Dynamics, and Quantum Efficiency. *J. Phys. Chem. C* **2017**, *121*, 14302–14311, doi:10.1021/acs.jpcc.7b01167.
9. Jung, M.; Scott, J.; Ng, Y.H.; Jiang, Y.; Amal, R. CuOx Dispersion and Reducibility on TiO_2 and Its Impact on Photocatalytic Hydrogen Evolution. *Int. J. Hydrog. Energy* **2014**, *39*, 12499–12506, doi:10.1016/j.ijhydene.2014.06.020.
10. Yoong, L.S.; Chong, F.K.; Dutta, B.K. Development of Copper-Doped TiO_2 Photocatalyst for Hydrogen Production under Visible Light. *Energy* **2009**, *34*, 1652–1661, doi:10.1016/j.energy.2009.07.024.
11. Yu, J.; Qi, L.; Jaroniec, M. Hydrogen Production by Photocatalytic Water Splitting over Pt/TiO_2 Nanosheets with Exposed (001) Facets. *J. Phys. Chem. C* **2010**, *114*, 13118–13125, doi:10.1021/jp104488b.
12. Zhang, J.; Yu, Z.; Gao, Z.; Ge, H.; Zhao, S.; Chen, C.; Chen, S.; Tong, X.; Wang, M.; Zheng, Z.; et al. Porous TiO_2 Nanotubes with Spatially Separated Platinum and CoOx Cocatalysts Produced by Atomic Layer Deposition for Photocatalytic Hydrogen Production. *Angew. Chem. Int. Ed.* **2017**, *56*, 816–820, doi:10.1002/anie.201611137.
13. Chen, X.; Xiao, S.; Wang, H.; Wang, W.; Cai, Y.; Li, G.; Qiao, M.; Zhu, J.; Li, H.; Zhang, D.; et al. MOFs Conferred with Transient Metal Centers for Enhanced Photocatalytic Activity. *Angew. Chem. Int. Ed.* **2020**, *59*, 17182–17186, doi:10.1002/anie.202002375.
